# Bromodomain-containing protein BRPF1 is a therapeutic target for liver cancer

**DOI:** 10.1038/s42003-021-02405-6

**Published:** 2021-07-20

**Authors:** Carol Lai-Hung Cheng, Felice Hoi-Ching Tsang, Lai Wei, Mengnuo Chen, Don Wai-Ching Chin, Jialing Shen, Cheuk-Ting Law, Derek Lee, Carmen Chak-Lui Wong, Irene Oi-Lin Ng, Chun-Ming Wong

**Affiliations:** 1 State Key Laboratory of Liver Research, The University of Hong Kong, Pok Fu Lam, Hong Kong; 2 Department of Pathology, Li Ka Shing Faculty of Medicine, The University of Hong Kong, Pok Fu Lam, Hong Kong

**Keywords:** Hepatocellular carcinoma, Epigenetics

## Abstract

Epigenetic deregulation plays an essential role in hepatocellular carcinoma (HCC) progression. Bromodomains are epigenetic “readers” of histone acetylation. Recently, bromodomain inhibitors have exhibited promising therapeutic potential for cancer treatment. Using transcriptome sequencing, we identified *BRPF1* (bromodomain and PHD finger containing 1) as the most significantly upregulated gene among the 43 bromodomain-containing genes in human HCC. BRPF1 upregulation was significantly associated with poor patient survival. Gene ablation or pharmacological inactivation of BRPF1 significantly attenuated HCC cell growth in vitro and in vivo. BRPF1 was involved in cell cycle progression, senescence and cancer stemness. Transcriptome sequencing revealed that BRPF1 is a master regulator controlling the expression of multiple key oncogenes, including *E2F2* and *EZH2*. We demonstrated that BRPF1 activated E2F2 and EZH2 expression by facilitating promoter H3K14 acetylation through MOZ/MORF complex. In conclusion, BRPF1 is frequently upregulated in human HCCs. Targeting BRPF1 may be an approach for HCC treatment.

## Abstract (web summary)

Cheng et al. suggest that BRPF1 plays an oncogenic role in hepatocellular carcinoma (HCC), by controlling histone acetylation in the promoter regions of *E2F2* and *EZH2*. Its high levels are correlated with poor survival in HCC patients, and targeting it might serve as a strategy for cancer treatment.

## Introduction

Hepatocellular carcinoma (HCC) is the most common type of liver cancer, accounting for ~80% of primary liver cancers^1^. HCC is the sixth most common cancer and the fifth deadliest cancer worldwide^2,3^. HCC is more commonly seen in men and has a higher incidence rate in developing countries^3^. HCC development is driven by the accumulation of genetic and epigenetic alterations. The major causes of HCC are hepatitis B (HBV) and hepatitis C (HCV) viral infections, cirrhosis, aflatoxin B1 ingestion, excessive alcohol consumption, and nonalcoholic fatty liver disease (NAFLD)^4^. Though the risk factors for HCC are relatively well defined, the diagnosis and prognosis of HCC are poor. Symptoms usually appear at the late stage of HCC, and metastasis is commonly observed in HCC patients^5^.

Liver transplantation or surgical resection is not applicable for patients diagnosed at the late stage of HCC^6^. Molecularly targeted therapy is the major method for the treatment of advanced HCC. Sorafenib has long been the only FDA-approved molecularly targeted therapy for HCC^7^. Recently, lenvatinib, regorafenib, and cabozantinib have also been approved for HCC treatment. These drugs are all multi-tyrosine kinase inhibitors, which are not specific and extend the survival rate only by a few months^8–10^. Drug resistance to sorafenib is commonly observed in HCC patients^11^. Immunotherapy maybe a promising new direction for HCC treatment. However, only a subset of HCC patients are responsive to anti-PD1 monoclonal antibodies^11^. Therefore, the discovery of potential therapeutic target and the development of new small molecule inhibitors with high specificity for HCC treatment are still urgently needed.

Bromodomain inhibitors represent a novel type of epigenetic drug and hold great promise for cancer therapy. Bromodomains are epigenetic “reader” domains that specifically recognize acetylated lysine residues on histones or nonhistone proteins^12^. Lysine acetylation is crucial for gene transcription, nucleosome assembly, protein–protein interaction and cellular signaling^13^. There are 43 bromodomain-containing proteins in humans, which are divided into eight subgroups depending on their structural similarities. Among these proteins, the members of the bromodomain and extra-terminal (BET) family have received considerable attention^14^. As registered in *ClinicalTrials.gov*, there are currently 23 ongoing clinical trials of bromodomain inhibitors at various phases. These trials largely focus on the therapeutic effect of BET inhibitors on human cancers, such as acute leukemia and prostate cancer, as well as metabolic disorders. However, the implications of other bromodomain-containing proteins in human carcinogenesis remain to be explored.

Herein, we identified bromodomain and PHD finger containing 1 (*BRPF1*) as the most significantly upregulated bromodomain-containing gene in human HCC. Overexpression of *BRPF1* was related to a poor survival rate in HCC patients. BRPF1 is an epigenetic reader protein containing a bromodomain, two PHD fingers and a PWWP domain. BRPF1 is mostly known as a component of the MOZ/MORF acetylation complex^15^. It has been reported that BRPF1 is critical for the formation of this complex as it links ING5 and MOZ/MORF together. BRPF1 also plays a role in regulating the histone acetyltransferase activity of MOZ/MORF^15,16^. It has been proposed that the bromodomain of BRPF1 may contribute to the chromatin binding and target specificity of the MOZ/MORF complex, wherein the catalytic subunit MOZ/MORF catalyzes H3K9, H3K14, and H3K23 acetylation to activate gene transcription^16–18^. BRPF1 is indispensable for embryonic development. Mouse genetic studies have shown that *BRPF1* knockout affects hematopoiesis as well as brain development and even causes early lethality^19,20^. Moreover, mutation of *BRPF1* causes intellectual disability and facial dysmorphisms in humans^21^. Recent studies demonstrated that *BRPF1* is recurrently mutated in adult Shh medulloblastoma^22^ and also critical for leukemogenesis associated with MOZ-TIF2 fusion^23^. Yet, the pathological and functional role of BRPF1 in cancer, especially liver carcinogenesis, is still largely unknown.

## Results

### Deregulation of bromodomain-containing genes in human HCC

**Fig. 1:**
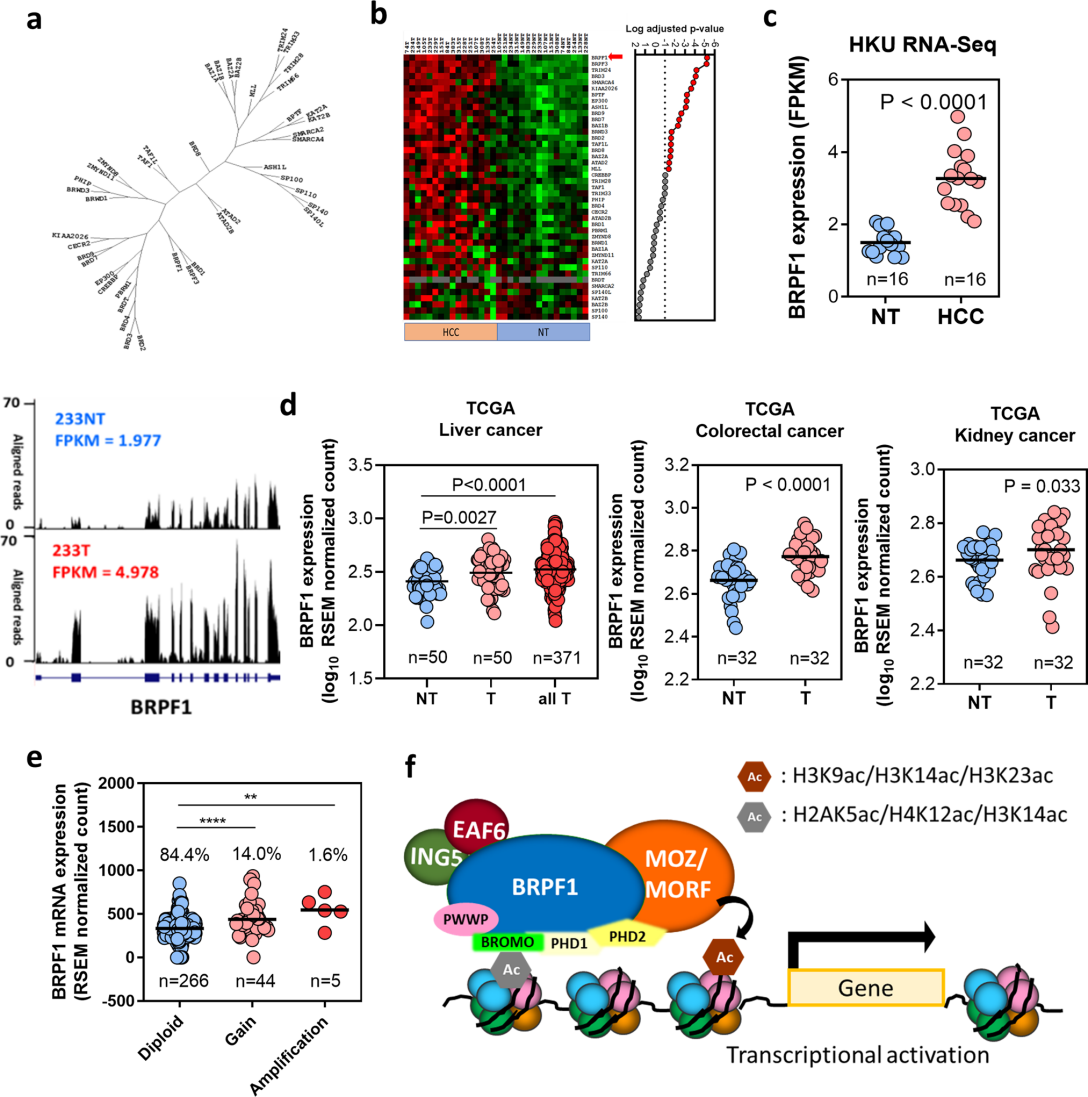
Frequent upregulation of BRPF1 in human HCC. **a** Phylogenetic tree of 43 human bromodomain-containing genes. **b** The expression of 43 bromodomain-containing genes in 16 pairs of human HCC tissues and their corresponding non-tumorous liver tissues by RNA-Seq. BRPF1 is at the top of the list. The *P*-values were calculated by paired *t*-test and adjusted for multiple testing. **c** Bromodomain and plant homeodomain (PHD) finger-containing protein (BRPF1) was highly upregulated in HCC. FPKM fragments per kilobase of transcript per million mapped reads. **d** BRPF1 expression was significantly upregulated in TCGA liver cancer, colorectal cancer, and kidney cancer cohorts. The *P*-values were calculated by paired *t*-test. T paired tumor, NT non-tumor samples, all T unpaired and paired tumors, RSEM RNA sequencing by expectation maximization. **e** *BRPF1* gene copy number gain and gene amplification were associated with increased BRPF1 mRNA expression in TCGA HCC cohort. The percentages in the graph represent the proportion of each condition among all tumors. **f** BRPF1 is a core subunit of the MOZ/MORF histone acetyltransferase complex, which mediates H3K9, H3K14, and H3K23 acetylation for gene activation. The BRPF1 protein possesses a unique combination of reader domains, which are a double PHD and zinc finger module (PZP), a bromodomain and a C-terminal PWWP domain. All data were compared by independent *t*-test unless indicated otherwise. **P < 0.01, ****P < 0.0001 vs. diploid as indicated.

Lysine acetylation is an abundant posttranslational modification in histone and nonhistone proteins. As an unique reader of protein acetylation, bromodomain-containing proteins are important for recognizing and responding to acetylation marks to regulate various cellular processes^24,25^. We hypothesized that deregulation of bromodomain-containing proteins might contribute to liver carcinogenesis. We therefore compared the expression levels of 43 human bromodomain-containing genes (as listed in the ChromoHub database^26^) in 16 pairs of HBV-associated primary HCC samples and their corresponding non-tumor (NT) liver samples by transcriptome sequencing (Fig. 1a, b). We found that about half of bromodomain-containing genes were highly upregulated in primary HCC. The results showed that upregulation of bromodomain-containing genes is a common phenomenon in human HCC, implying that the demand for reading acetylation marks is increased for HCC cell growth.

### Frequent upregulation of BRPF1 in human HCC

In the above transcriptome analysis, we identified *BRPF1* as the most significantly upregulated bromodomain-containing gene in HCC, according to the statistical significance (Rank 1) and expression change (Rank 10) (Fig. 1b, c). In fact, not just in the in-house samples, *BRPF1* was also upregulated in HCC, colorectal cancer and kidney cancer from TCGA database (Fig. 1d). The BRPF1 upregulation in TCGA HCC cohort was relatively modest compared to that in the in-house samples. However, the in-house data comes from Chinese patients associated with HBV-infection, while TCGA database contains samples with different aetiological backgrounds and ethnicities. The upregulation of BRPF1 expression in HCC tumor samples may be affected by different aetiological factors. After all, these expression data from TCGA database reinforced our initial observation in the RNA-seq analysis, suggesting that BRPF1 may be essential for cancer development. We found that copy number gain or amplification of the *BRPF1* gene located on chromosome 3p25 was frequent in human HCC. In addition, HCC with *BRPF1* gene copy number gain/amplification had higher BRPF1 mRNA expression than other HCC, suggesting that gain of gene copy number may directly contribute to BRPF1 upregulation in human HCC (Fig. 1e). BRPF1, an epigenetic reader with multiple reader domains (Supplementary Fig. S1), is a major component of the MOZ/MORF acetyltransferase complex (Fig. 1f). We thus speculated that activation of the BRPF1/MOZ/MORF complex may play an important role in liver carcinogenesis.

### Clinicopathological relevance of BRPF1 upregulation in human HCC

**Fig. 2:**
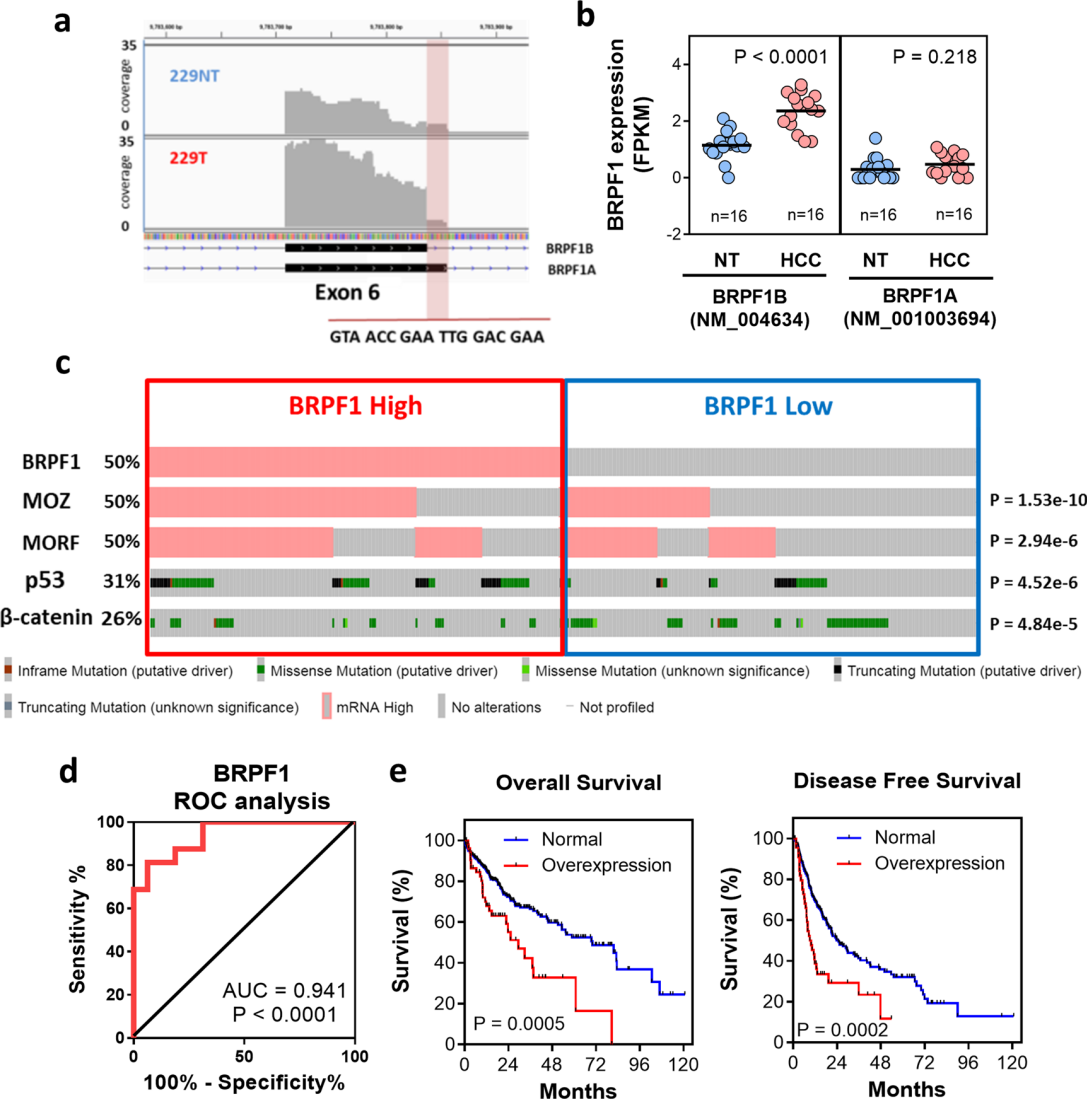
Clinical relevance of BRPF1 upregulation in HCC. **a** Raw data of aligned sequencing reads on the exon 6 of *BRPF1* gene in a representative HCC case. *BRPF1* gene expresses two isoforms, namely BRPF1A and BRPF1B. BRPF1A utilizes an alternative splice site at the exon 6 and generates an inactive protein with 6 extra amino acids, while BRPF1B encodes a functional protein. **b** Only BRPF1B isoform was significantly upregulated in HCC samples from HKU cohort. The *P*-values were calculated by paired *t*-test. **c** High BRPF1 expression was significantly correlated with high MOZ and MORF expression in TCGA HCC cohort. In addition, high BRPF1 expression (median cut-off) was significantly concurrent with the *TP53* mutation but mutually exclusive with the *CTNNB1* mutation. The *P*-values were calculated by *χ*^2^ test. **d** ROC analysis in HKU cohort demonstrating that BRPF1 expression is a potential tissue biomarker for HCC detection. *n* = 16 **e** High BRPF1 overexpression was associated with poorer overall (*n* = 357) and disease-free survival (*n* = 306) of HCC patients in TCGA HCC cohort. The survival rates were compared by log rank test.

*BRPF1* has two alternative splicing isoforms (BRPF1A and BRPF1B) (Fig. 2a). BRPF1B encodes functional BRPF1 protein, while BRPF1A uses an alternative splice site at exon 6, leading to an insertion of 6 extra amino acids. This insertion results in a structural change in the bromodomain that hinders its binding with acetylated lysine residue^27^, which causes BRPF1A to become a functionally inactive isoform. To distinguish these two BRPF1 isoforms, we reanalyzed our RNA-seq raw data with an isoform-specific algorithm. We found that BRPF1B was the predominant isoform in HCC samples, accounting for ~80% of the BRPF1 transcripts. This isoform-specific analysis also confirmed that only the functional BRPF1B isoform was significantly upregulated in human HCC, while the expression of BRPF1A remained unchanged (Fig. 2b). These findings indicated that upregulation of functional BRPF1 may play a role in HCC. Moreover, we observed a positive correlation between the expression level of BRPF1 and those of MOZ and MORF (Fig. 2c), suggesting a coordinated upregulation of the subunits of MOZ/MORF complex in human HCC. To delineate the potential implications of BRPF1 overexpression in human HCC, the effects of BRPF1 mRNA expression on several clinicopathological features were examined. *TP53* and *CTNNB1* mutations are both major cancer driver events in human HCC^28^. We noted that high BRPF1 expression (median cut-off) was concurrent with *TP53* mutation but intriguingly mutually exclusive with *CTNNB1* mutation (Fig. 2c). ROC analysis showed that BRPF1 expression can distinguish HCC from NT samples with high sensitivity and specificity (AUC = 0.941), indicating that BRPF1 could be a potential tissue biomarker for human HCC detection (Fig. 2d). We also found that high BRPF1 expression was significantly associated with a poorer overall survival rate and disease-free survival rate in TCGA HCC cohort (Fig. 2e). The above findings suggested that BRPF1 upregulation has significant clinicopathological implications in HCC tumorigenicity.

### Overexpression of SP1 transcription factor caused BRPF1 upregulation in HCC

**Fig. 3:**
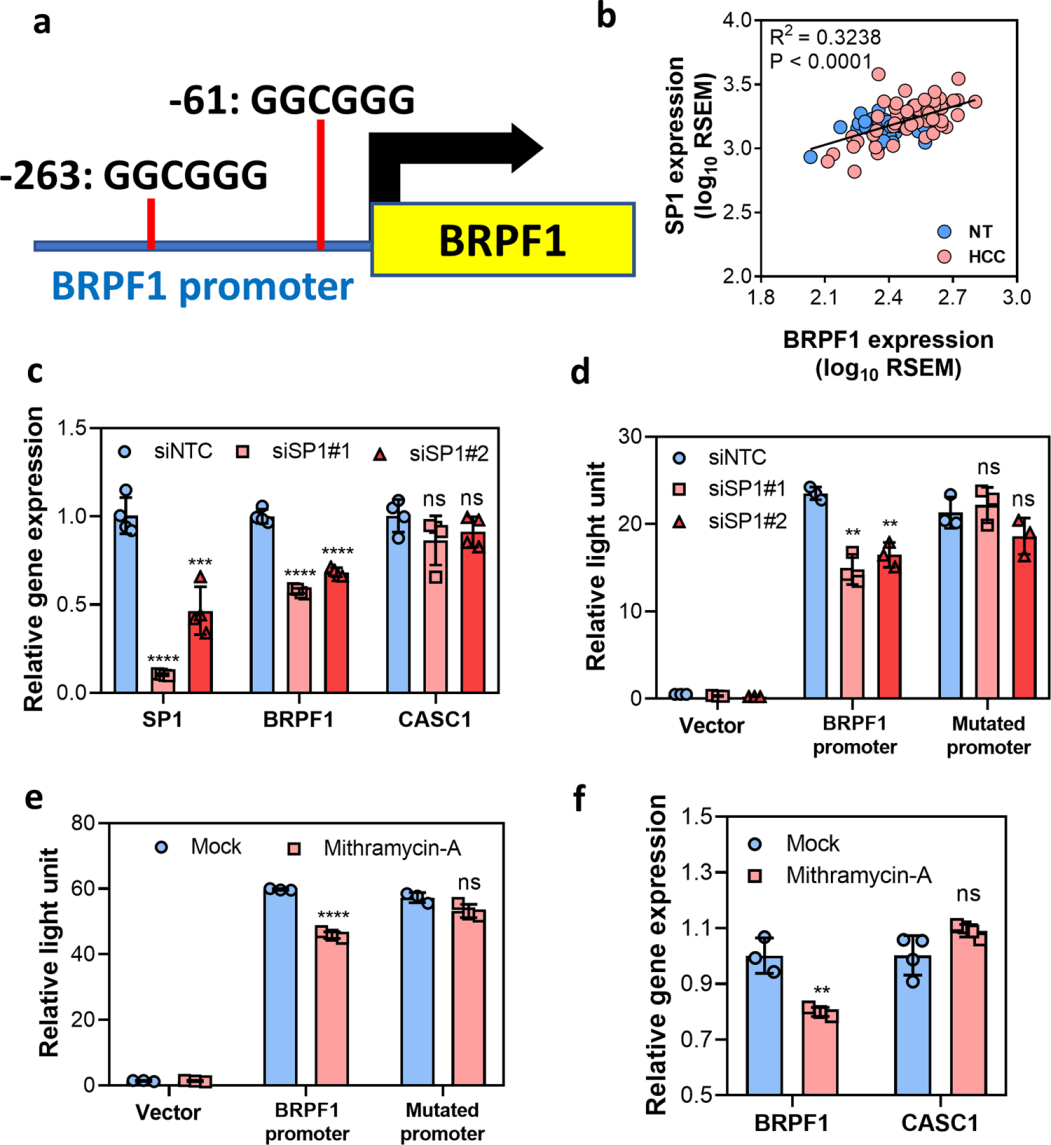
SP1 upregulation facilitated the increase in BRPF1 expression. **a** The *BRPF1* promoter contains two SP1 putative binding sites. **b** The expression of SP1 was positively correlated with the expression of BRPF1 in TCGA HCC cohort (*R*^2^ = 0.3238, *P* < 0.0001, linear regression, *n* = 50). **c** Inactivation of SP1 by siSP1 reduced the mRNA expression of BRPF1 and SP1 in HEK293T cells, while the expression of CASC1, a non-SP1 target, remained unchanged. **d** Treatment with siSP1 decreased *BRPF*1 promoter activity, but not the activity of SP1 binding site mutated BRPF1 promoter in HEK293T cells. **e** 20 nM mithramycin A, a SP1 inhibitor, reduced *BRPF1* promoter activity in HEK293T cells after 48-h treatment. **f** 20 nM mithramycin A reduced the mRNA expression of BRPF1 in MHCC97L after 48-h treatment. Error bars indicate mean ± SD. Data were compared by independent *t*-test unless indicated otherwise. Results were repeated at least three times. **P < 0.01, ***P < 0.001, ****P < 0.0001, ns not significant vs. mock or siNTC as indicated.

The initial observation of BRPF1 upregulation in clinical human HCC led to further investigation on the underlying mechanism of the upstream regulatory factor of BRPF1 expression. In silico analysis (PROMO 3.0) identified two SP1 putative binding sites with a consensus sequence GGCGGG in the *BRPF1* promoter (Fig. 3a). In addition, SP1 was highly upregulated in liver cancer, and the expression of SP1 was positively correlated with BRPF1 expression (Fig. 3b). SP1 is a transcription factor known to regulate the expression of multiple oncogenes related to cell proliferation, cell cycle, and metastasis, thus contributing to cancer development including HCC^29^. Hence, we hypothesized that *BRPF1* was one of the oncogenes regulated by SP1. To confirm the regulatory role of SP1 on *BRPF1* promoter activity, a luciferase reporter assay was performed. Inactivation of SP1 by siRNA (siSP1#1 and siSP1#2) reduced the mRNA expression of BRPF1 and SP1, while the expression of SP1 non-target gene, CASC1, remained unchanged (Fig. 3c). More importantly, silencing SP1 suppressed *BRPF1* promoter activity, but not the SP1 binding site mutated *BRPF1* promoter (Fig. 3d and Supplementary Fig. S2). As expected, the SP1 inhibitor mithramycin A also lowered *BRPF1* promoter activity (Fig. 3e). Upon 48-h treatment with mithramycin A, the mRNA expression of BRPF1 in MHCC97L was significantly reduced (Fig. 3f). The above findings collectively suggested that overexpression of SP1 contributed to BRPF1 upregulation in human HCC.

### CRISPR knockout of *BRPF1* inhibited HCC growth in vivo

**Fig. 4:**
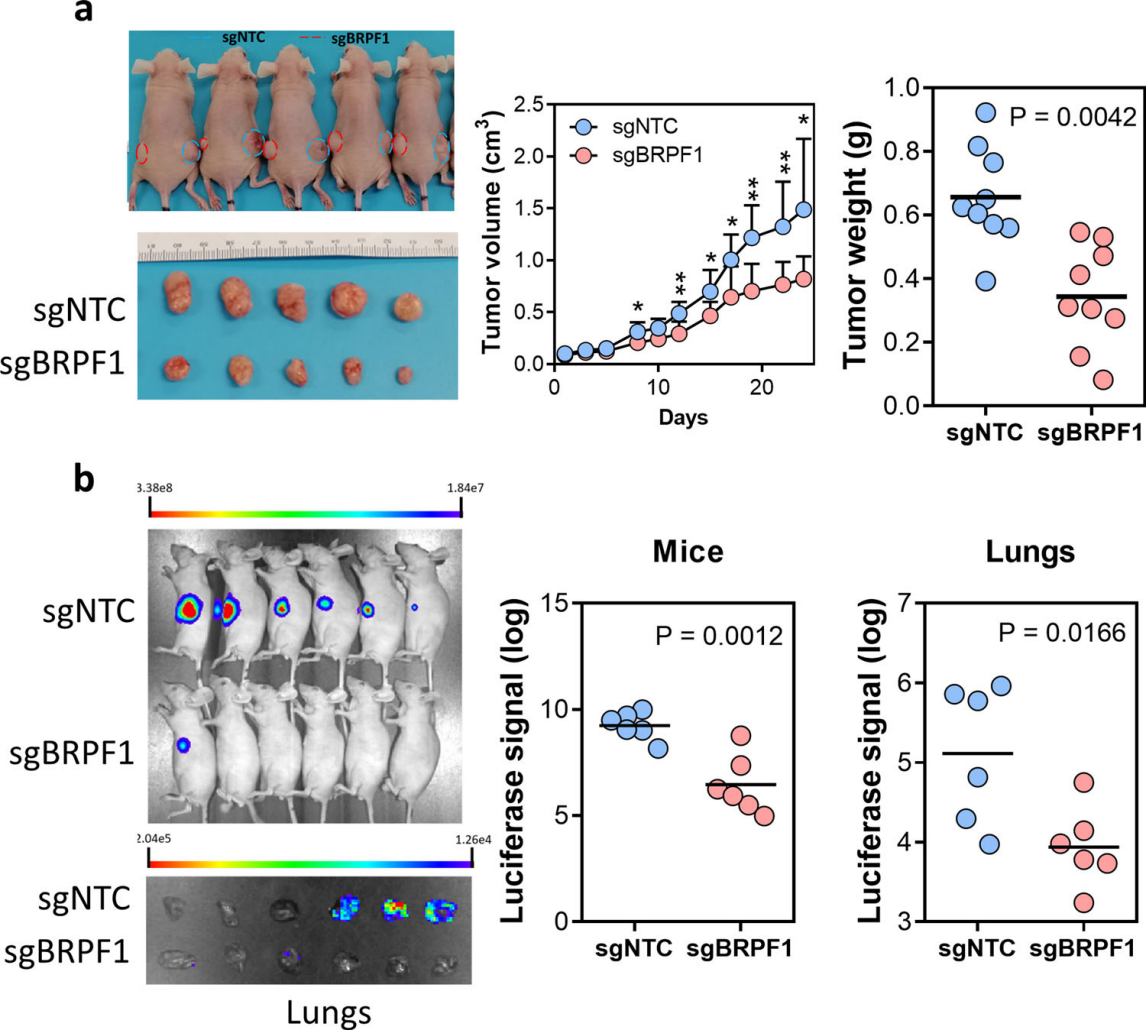
Knockout of *BRPF1* suppressed HCC growth in vivo. **a** *BRPF1* knockout reduced subcutaneous tumor growth in nude mice. The tumor volumes and tumor weights between two groups were compared by paired *t*-test. *n* = 9 **b** Knockout of *BRPF1* dramatically suppressed HCC orthotopic tumor growth in the liver and lung metastasis in nude mice. Data were compared by independent *t*-test. *n* = 6 *P < 0.05, **P < 0.01 vs. sgNTC as indicated.

HCC cell lines generally showed increased BRPF1 expression compared to primary HCC and NT liver samples (Supplementary Fig. S3A). Among all HCC cell lines, MHCC97L, Huh-7, and Hep3B are the top three cell lines showing the highest expression of BRPF1. Considering that MHCC97L have a greater tumor-formation ability in nude mice, MHCC97L was chosen for the major cell model for subsequent mechanistic study and in vivo experiment. To dissect the pathological role of BRPF1 in human HCC, we employed two shRNAs (shBRPF1#1 and shBRPF1#2) to knockdown BRPF1 separately, which retarded the cell proliferation and colony formation (Supplementary Fig. S4A–C). We also used another approach to inhibit BRPF1 expression by CRISPR/Cas9 genome editing system with an sgRNA sequence. Since an effective antibody for BRPF1 is unavailable, we validated the knockout efficiency by Sanger sequencing and tide analysis (Supplementary Fig. S3B, C), which showed a successful induction of indel on *BRPF1* gene by the CRISPR system. To verify the oncogenic role of BRPF1 in HCC development, a subcutaneous injection experiment was performed. We found that *BRPF1* knockout in MHCC97L cells significantly reduced subcutaneous tumor growth in nude mice (Fig. 4a). As the tumor microenvironment is crucial for tumor growth, we further performed orthotopic xenograft experiment to allow HCC tumor growth in its native microenvironment. The results showed that *BRPF1* knockout markedly abolished HCC tumorigenicity (Fig. 4b). Lung metastasis was also suppressed by *BRPF1* knockout, as evidenced by the ex vivo bioluminescence imaging of the lungs from tumor-bearing mice (Fig. 4b). Consistent with the in vivo finding, *BRPF1* knockout cells demonstrated a lower migration rate (Supplementary Fig. S3D). To further demonstrate *BRPF1* is an oncogene, we established BRPF1 overexpressing model by CRISPR/dCas9-SAM system in Hep3B which has a lower BRPF1 expression compared to MHCC97L (Supplementary Fig. S3A and S4D). BRPF1 overexpression increased cell proliferation (Supplementary Fig. S4E). The above results showed that BRPF1 plays an oncogenic role in HCC development.

### GSK5959, a BRPF1-specific inhibitor, inhibited HCC growth

**Fig. 5:**
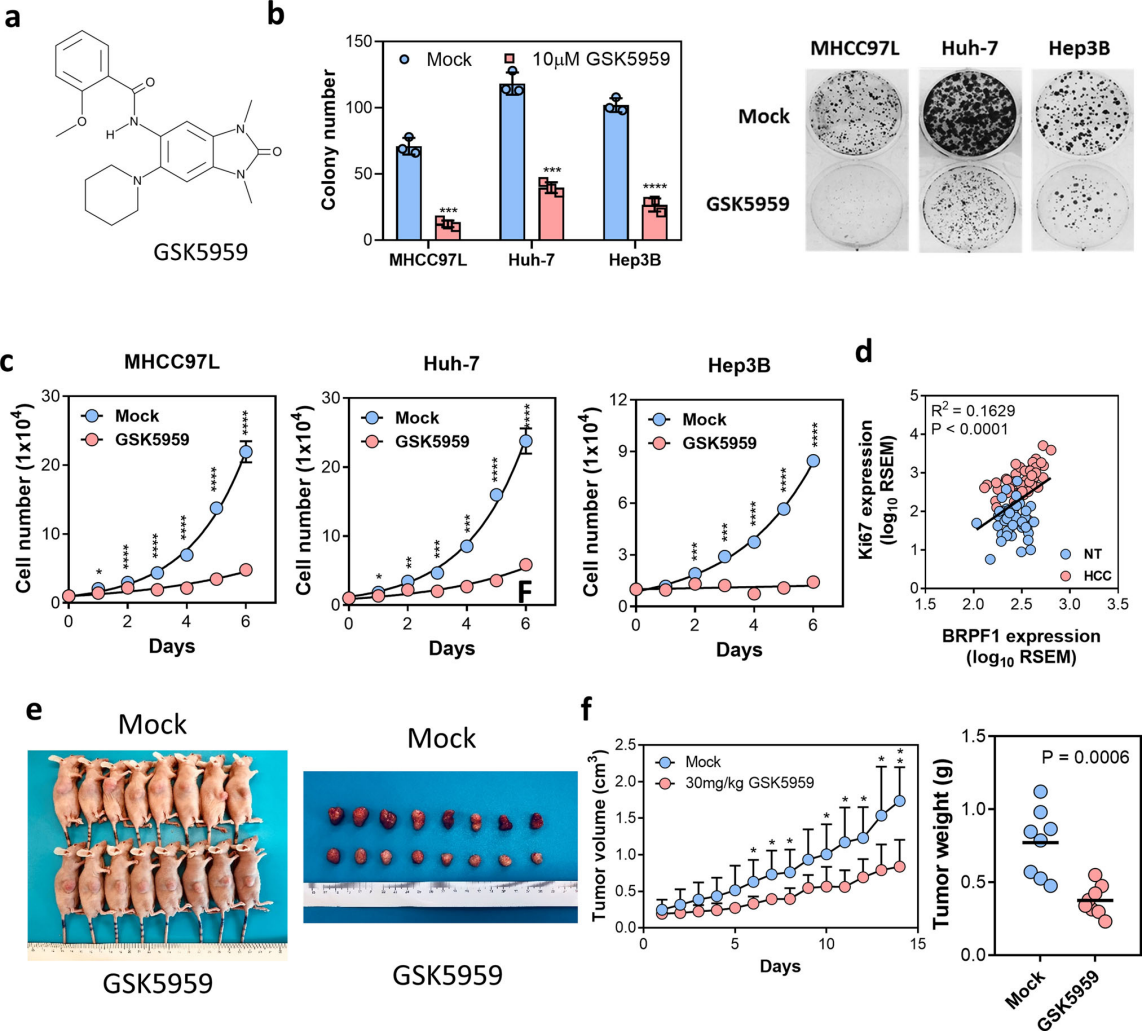
GSK5959 inhibited HCC development in vitro and in vivo. **a** GSK5959 is a BRPF1-specific inhibitor. **b**, **c** Treatment with 10 μM GSK5959 significantly reduced cell colony formation and cell proliferation in multiple HCC cell lines. **d** BRPF1 expression was positively correlated with Ki67 expression in TCGA HCC cohort (*R*^2^ = 0.1629, *P* < 0.0001, linear regression). **e** Displays of mice treated with mock (DMSO) or GSK5959 (30 mg/kg) via intraperitoneal injection once a day for 2 weeks and isolated subcutaneous tumors in the order of decreasing size. **f** The tumor volume and weight in the GSK5959 treatment group were significantly lower than those in the control group. *n* = 8 Error bars indicate mean ± SD. Data were compared by independent *t*-test unless indicated otherwise. Results were repeated at least three times. *P < 0.05, **P < 0.01, ***P < 0.001, ****P < 0.0001 vs. mock as indicated.

Since our initial data suggested that BRPF1 may be a potential therapeutic target to suppress HCC growth, we investigated the therapeutic potential of GSK5959, a BRPF1-specific inhibitor, in HCC. (Fig. 5a). The biological effects of GSK5959 in cellular or in vivo studies have not been reported. Here we demonstrated that BRPF1 inhibition by GSK5959 suppressed colony formation and cell proliferation in several HCC cell lines with high expression of BRPF1 (Fig. 5b, c). The expression of Ki67, a marker for proliferation, was positively correlated with BRPF1 expression in TCGA HCC cohort, further supporting the role of BRPF1 in HCC proliferation (Fig. 5d). Yet, BRPF1 inhibition had no significant effect on apoptosis (Supplementary Fig. S5). To examine whether pharmacological inactivation of BRPF1 could suppress HCC growth in vivo, we treated tumor-bearing nude mice with GSK5959 (30 mg/kg/day via intraperitoneal injection) for 2 weeks. We found that GSK5959 significantly inhibited subcutaneous tumor growth without causing observable toxicity (Fig. 5e, f). These findings suggest that GSK5959 is a potential therapeutic drug for HCC treatment.

### BRPF1 is related to liver cancer stem cell properties

**Fig. 6:**
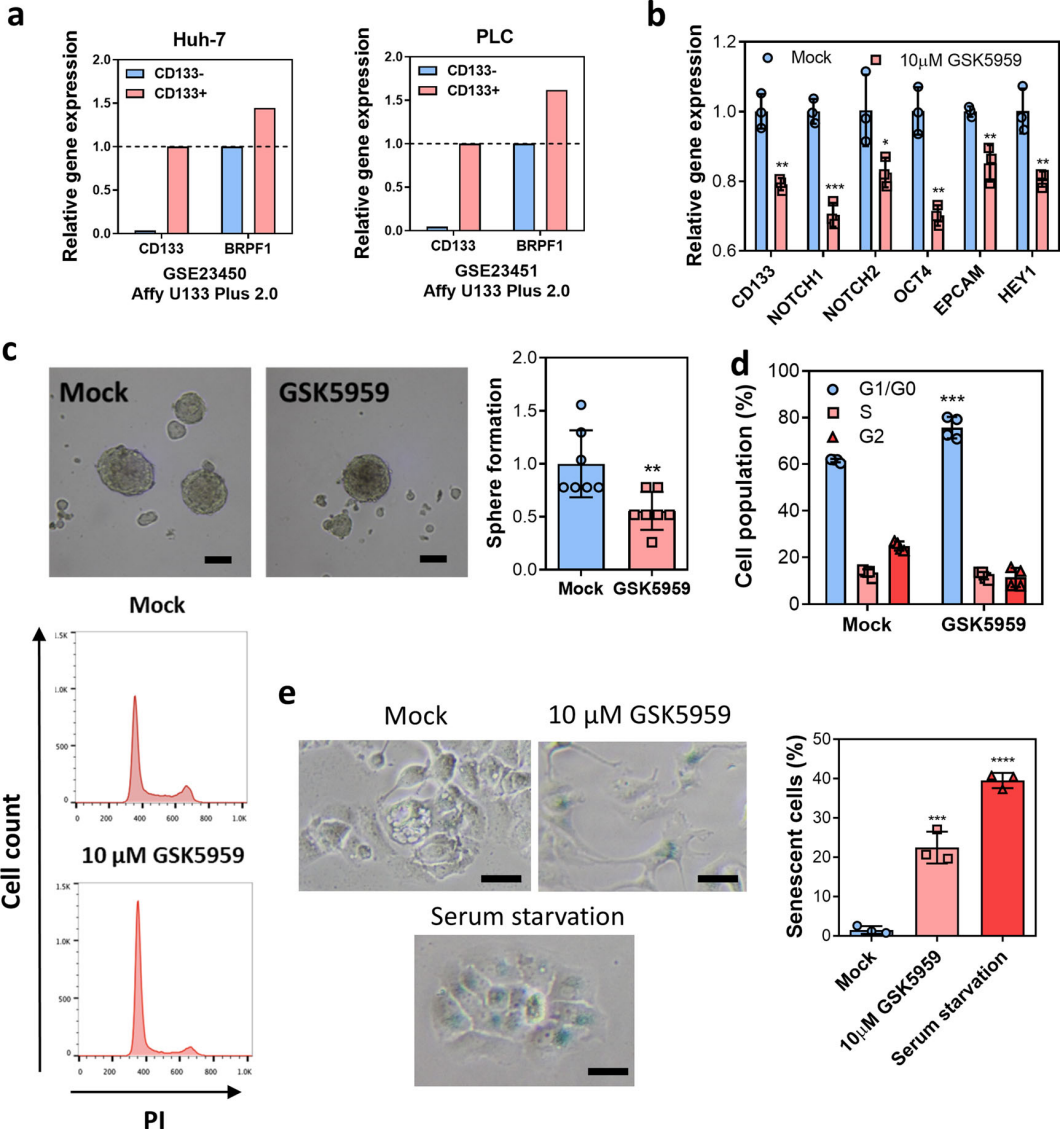
BRPF1 is related to liver cancer stem cell properties. **a** Upregulation of BRPF1 in CD133 + positive liver cancer stem cells compared to CD133- Huh-7 and PLC cells. cDNA microarray data were extracted from Gene Expression Omnibus: GSE23450 and GSE23451. **b** GSK5959 reduced the expression of stem cell factors in Huh-7 cells after 24-h treatment. **c** In all, 10 μM GSK5959 treatment for a week significantly reduced the sphere formation ability of Huh-7 cells in nonadherent culture condition (Scale bar: 0.1 mm). **d** 10 μM GSK5959 induced cell cycle G1 arrest in MHCC97L cells after 48-h treatment. **e** 10 μM GSK5959 induced cellular senescence in MHCC97L cells after 5-day treatment, as shown by the β-galactosidase assay (Scale bar: 0.3 mm). Serum-starved cells served as a positive control. Error bars indicate mean ± SD. Data were compared by independent *t*-test. Results were repeated at least three times. *P < 0.05, **P < 0.01, ***P < 0.001, ****P < 0.0001 vs. mock as indicated.

The functional role of BRPF1 in cancer development remains largely unknown. To elucidate the mechanism by which BRPF1 contributes to carcinogenesis, various cancer hallmarks were examined. Cancer stemness is considered as an important feature in cancer and one of the main causes of drug resistance and remission^30^. cDNA microarray analysis of PLC and Huh-7 cells revealed that BRPF1 expression was upregulated in CD133 + liver cancer stem cells when compared to its CD133- counterparts, indicating that BRPF1 may be associated with cancer stemness (Fig. 6a). We then further detected the expression of stem cell factors involved in HCC. A panel of stemness genes, such as NOTCH1, OCT4, and EPCAM, were downregulated upon BRPF1 inhibition by GSK5959 in Huh-7 (Fig. 6b). Interestingly, the expression of CD133 was reduced upon GSK5959 treatment, which echoed the microarray data and implied that BRPF1 regulates the expression of CD133. We also demonstrated that GSK5959 reduced sphere formation in Huh-7 cells which have a greater stemness potential and more readily form spheres compared to other HCC cell lines (Fig. 6c). The above findings suggested that BRPF1 is related to liver cancer stem cell properties.

To study the role of BRPF1 in regulating cell proliferation, we performed cell cycle analysis by flow cytometry. GSK5959 induced G1 cell cycle arrest in MHCC97L cells, which may partially explain the suppressive effect of BRPF1 inhibition on the proliferation rate (Fig. 6d). GSK5959 also induced cellular senescence, as shown by β-galactosidase staining (Fig. 6e). Taken together, our data demonstrated that GSK5959 is a potential small molecule drug for HCC treatment by inhibiting HCC cell growth and cancer stem cell properties.

### E2F2 and EZH2 are the downstream targets of BRPF1

**Fig. 7:**
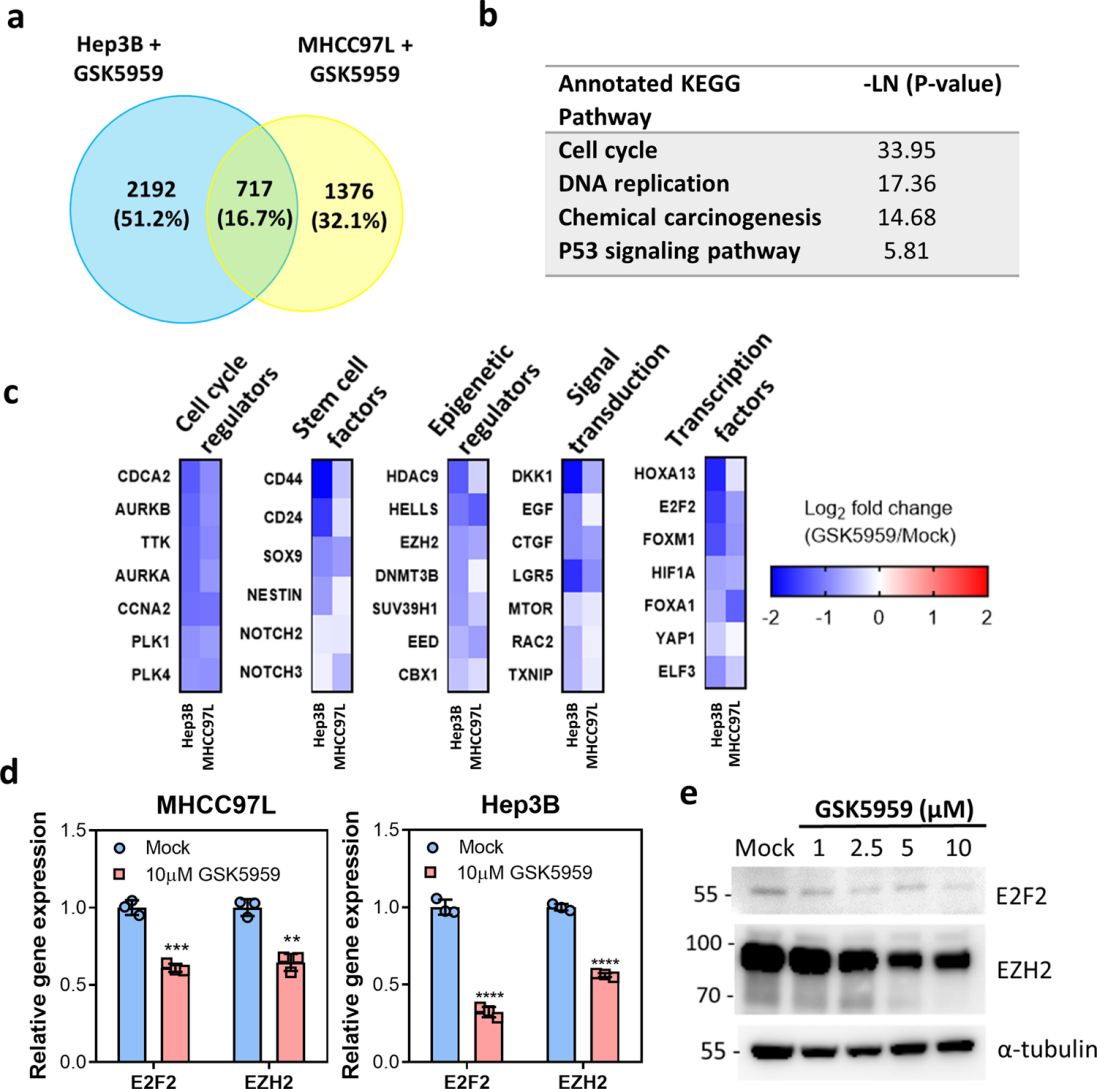
RNA-seq data identified E2F2 and EZH2 as the downstream targets of BRPF1. **a** RNA-seq identified differentially expressed genes in MHCC97L and Hep3B cells treated with 10 μM GSK5959 for 24 h. **b** KEGG pathway analysis of the common downregulated genes from the RNA-seq results. **c** GSK5959 treatment selectively led to reduced expression of key oncogenes essential for cancer growth and progression. **d** The mRNA expression levels of E2F2 and EZH2 were downregulated by GSK5959 treatment after 24 h in MHCC97L and Hep3B. **e** GSK5959 inhibited the protein levels of E2F2 and EZH2 in a dose-dependent manner after 5-day treatment. Error bars indicate mean ± SD. Data were compared by independent *t*-test. Results were repeated at least three times. **P < 0.01, ***P < 0.001, ****P < 0.0001 vs mock as indicated.

To understand the molecular mechanism by which BRPF1 contributes to HCC tumorigenicity and identify BRPF1 downstream targets, we analyzed the transcriptome changes in MHCC97L and Hep3B cells upon GSK5959 treatment by RNA-seq. A total of 717 genes were found to be commonly downregulated in both MHCC97L and Hep3B cells (Fig. 7a). KEGG pathway analysis showed that these common downregulated genes were associated with cell cycle, DNA replication, chemical carcinogenesis, and p53 signaling pathway (Fig. 7b), highlighting the relevance of BRPF1 in cancer development. Further examination of the RNA-seq data revealed that BRPF1 is a master regulator on the expression of key oncogenes essential for cell cycle progression, cancer stemness, epigenetic regulation, and signal transduction (Fig. 7c). Among the common downregulated genes, the expression of E2F2 and EZH2 was validated by qRT-PCR (Fig. 7d). The mRNA and protein expression of E2F2 and EZH2 was reduced by GSK5959 treatment in a dose-dependent manner, further confirming the effect of BRPF1 inhibition on the regulation of E2F2 and EZH2 expression (Fig. 7e and Supplementary Fig. S6A). In parallel, we used two pan-BRPF inhibitors, OF-1 and NI-57. Consistent with the finding in GSK5959 treatment, the expression of E2F2 and EZH2 was suppressed upon OF-1 or NI-57 treatment in a dose-dependent manner (Supplementary Fig. S6B). In addition, both inhibitors suppressed HCC cell proliferation (Supplementary Fig. S6C) and induced cellular senescence (Supplementary Fig. S6D).

**Fig. 8:**
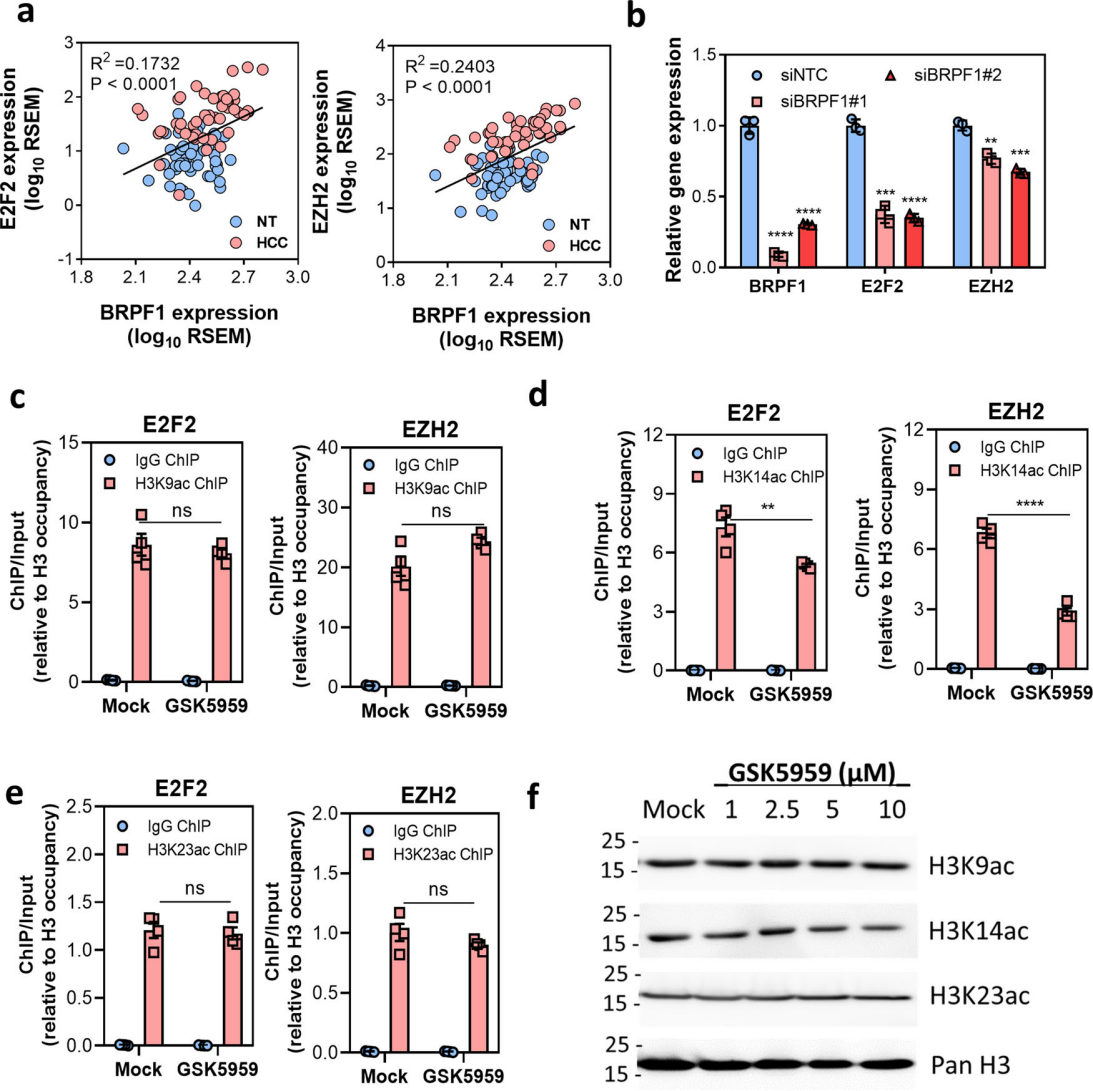
BRPF1 regulated the expression of downstream targets through epigenetic modification. **a** BRPF1 expression was positively correlated with the expression of E2F2 (*R*^2^ = 0.1732, *P* < 0.0001, linear regression) and EZH2 (*R*^2^ = 0.2403, *P* < 0.0001, linear regression) respectively in TCGA HCC cohort. *n* = 50 **b** The mRNA expression of E2F2 and EZH2 was reduced in MHCC97L cells siRNA-mediated BRPF1 knockdown. **c** H3K9 acetylation levels at the promoters of E2F2 and EZH2 remained unchanged after 48-h of GSK5959 treatment in MHCC97L cells. **d** H3K14 acetylation levels at the promoters of E2F2 and EZH2 were reduced upon 48-h of GSK5959 treatment in MHCC97L cells. **e** H3K23 acetylation level at the promoters of E2F2 and EZH2 remained unchanged after 48-h of GSK5959 treatment in MHCC97L cells. All acetylation levels were normalized by histone H3 occupancy level. **f** GSK5959 had no effect on the global levels of H3K9, H3K14, and H3K23 acetylation in MHCC97L cells after 5-days treatment. Error bars indicate mean ± SD. Data were compared by independent *t-*test unless indicated otherwise. Results were repeated at least three times. **P < 0.01, ***P < 0.001, ****P < 0.0001, ns not significant vs siNTC or IgG ChIP as indicated.

*E2F2* and *EZH2* are two well-known oncogenes involved in HCC development and upregulated in HCC (Supplementary Fig. S7). The expression of E2F2 and EZH2 was positively correlated with BRPF1 expression in TCGA cohort (Fig. 8a). CRISPR knockout of *BRPF1* reduced the expression of E2F2 and EZH2 in the orthotopic liver tumors (Supplementary Fig. S8A). BRPF1 knockdown by siRNA or shRNA also significantly decreased the expression of E2F2 and EZH2 in MHCC97L (Fig. 8b and Supplementary Fig. S8B, C), while the expression of E2F2 and EZH2 was upregulated in BRPF1 overexpressing Hep3B cells (Supplementary Fig. S8D). BRPF1 is a component of the MOZ/MORF acetyltransferase complex. We hypothesized that BRPF1 regulated the expression of E2F2 and EZH2 through MOZ/MORF-mediated histone acetylation. We then performed ChIP assay to assess the local acetylation levels at the promoters of *E2F2* and *EZH2* and normalize the acetylation levels by respective histone H3 occupancy. Interestingly, GSK5959 inhibited H3K14 acetylation but not H3K9 or H3K23 acetylation at both promoters (Fig. 8c–e). In addition, GSK5959 treatment did not alter the global H3K9, H3K14, or H3K23 acetylation levels (Fig. 8f). The results collectively suggested that BRPF1 specifically regulates the histone modification of its downstream genes rather than the global histone acetylation levels. Our findings shed light on the mechanism by which BRPF1 upregulation contributes to HCC progression.

### MOZ/MORF inhibition suppressed BRPF1 downstream targets, E2F2 and EZH2

**Fig. 9:**
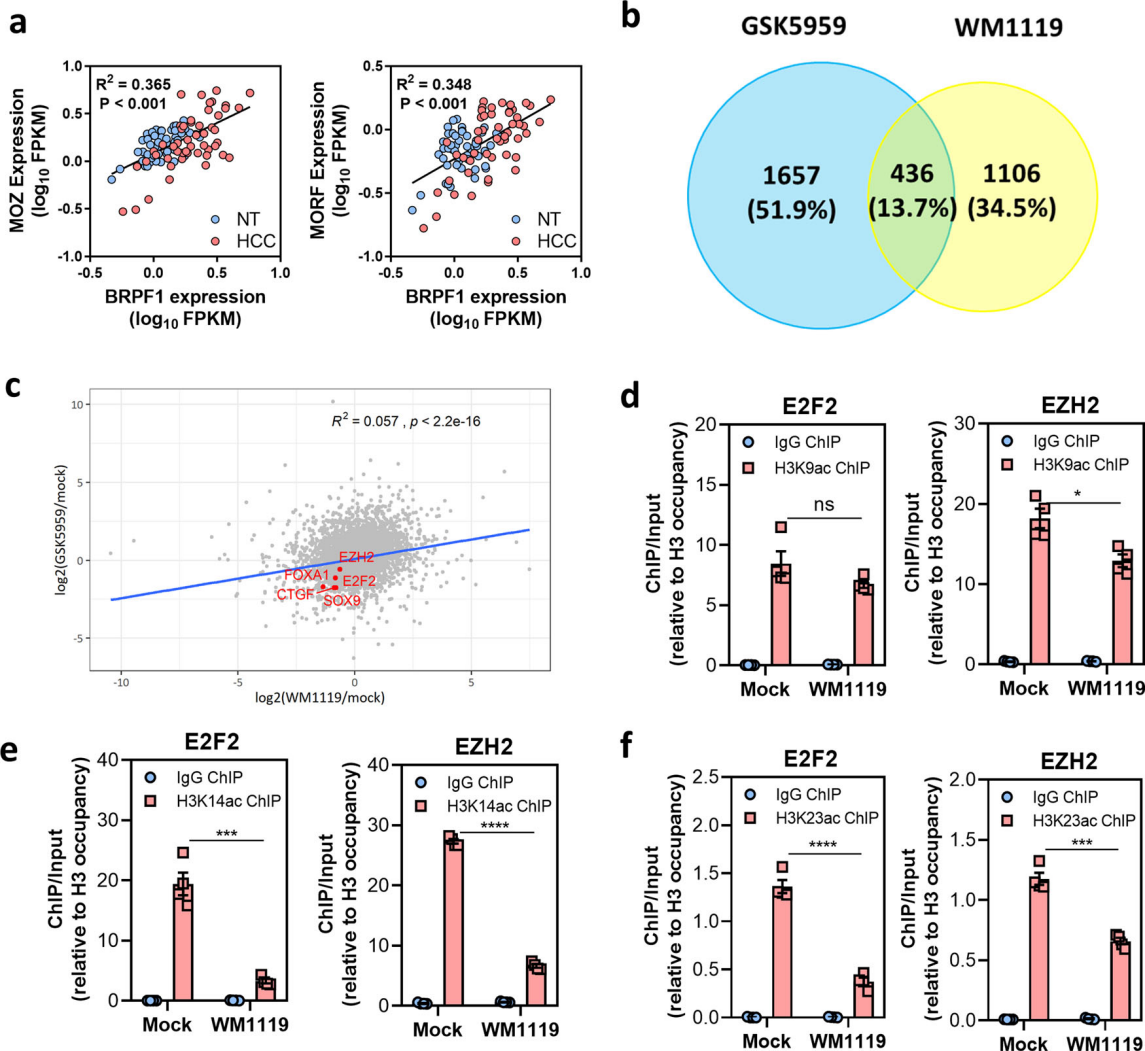
MOZ/MORF inhibition suppressed the expression of the BRPF1 downstream targets *E2F2* and *EZH2*. **a** BRPF1 expression was positively correlated with MOZ (*R*^2^ = 0.365, *P* < 0.001, linear regression) and MORF expression (*R*^2^ = 0.348, *P* < 0.001, linear regression) in TCGA HCC cohort. *n* = 50 **b** RNA-seq identified 436 common differentially expressed genes in MHCC97L upon GSK5959 or WM1119 treatment. **c** Differential gene expression upon GSK5959 or WM1119 treatment in MHCC97L was significantly correlated. BRPF1 and MOZ co-regulated the expression of several oncogenes, including *E2F2* and *EZH2*. **d** H3K9 acetylation level at the promoter of *E2F2* remained unchanged while the level at the promoter of *EZH2* was reduced upon 100 μM WM1119 treatment for 72 h. **e** H3K14 and **f** H3K23 acetylation levels at the promoters of *E2F2* and *EZH2* were reduced upon 100 μM WM1119 treatment for 72 h. All acetylation levels were normalized by histone H3 occupancy level. Error bars indicate mean ± SD. Data were compared by independent *t*-test unless indicated otherwise. Results were repeated at least three times. *P < 0.05, ***P < 0.001, ****P < 0.0001, ns not significant vs IgG ChIP as indicated.

BRPF1 is a subunit of the MOZ/MORF acetyltransferase complex. BRPF1 expression was positively correlated with MOZ and MORF expression in HCC (Fig. 9a). MOZ or MORF inhibition by their respective gene-specific siRNAs or MOZ/MORF inhibitor called WM1119 led to a reduction on the expression of E2F2 and EZH2 (Supplementary Fig. S9A, B) in both MHCC97L and Hep3B. To further validate the association of MOZ/MORF complex and BRPF1 downstream target, we performed RNA-seq on MHCC97L cells treated with either WM1119 or EPZ6438, which is a EZH2-specific inhibitor. EZH2 inhibition by EPZ6438 reduced cell proliferation (Supplementary Fig. S9C). There were 436 common differentially expressed genes upon GSK5959 or WM1119 treatment, which accounted for 13.7% (Fig. 9b). Moreover, the differentially expressed genes upon GSK5959 or WM1119 treatment were significantly correlated while MOZ/MORF and BRPF1 co-regulated the expression of multiple oncogenes, including *E2F2*, *EZH2*, *SOX9*, and *FOXA1* (Fig. 9c). We selected the upregulated genes (fold change>2) upon EPZ6438 treatment to make a EZH2 target gene set, then performed gene set enrichment analysis. The analysis revealed that differentially expressed genes in GSK5959 and WM1119 treated MHCC97L were enriched for EZH2 target genes (Supplementary Fig. S9D). The above results collectively showed that MOZ/MORF regulated the downstream targets of BRPF1.

We then hypothesized that BRPF1 regulated the H3K14 acetylation at the promoters of its downstream targets through the acetyltransferase activity of MOZ/MORF. Intriguingly, after normalization to histone H3 occupancy, the ChIP assay showed that WM1119 inhibited H3K9, H3K14, and H3K23 acetylation at the promoter of *EZH2* and reduced H3K14 and H3K23 acetylation at the *E2F2* promoter (Fig. 9d–f). We then examined the global effect on the histone acetylation upon WM1119 treatment by western blotting, which showed a general decrease on all three histone marks in different degrees (Supplementary Fig. S10A). In addition, pharmacological inactivation of BRPF1 by GSK5959 partially reduced the MOZ genomic localization at the promoters of *E2F2* and* EZH2* (Supplementary Fig. S10B). Taken altogether, our data demonstrated that MOZ/MORF complex regulates the expression of *E2F2* and *EZH2* by modifying the H3K14 acetylation at their promoters.

## Discussion

Cancers are caused not only by gene mutations but also by epigenetic aberrations^31^. Since epigenetic modifications are reversible and dynamic, epigenetic regulators have become attractive drug targets for human cancers^32^. Small molecule inhibitors, such as histone deacetylase inhibitors and DNA methyltransferase inhibitors, have already been approved by FDA for the treatment of multiple types of cancer^33^. Currently, FDA-approved small molecule drugs for advanced HCC are limited to several multi-tyrosine kinase inhibitors, such as sorafenib and lenvatinib. Unfortunately, they only extend the patient survival to a few months on average, while drug resistance frequently compromises the clinical outcome of these inhibitors^11^. They also cause substantial side effects in the patients due to low specificity^34^. Therefore, development of new small molecule drugs with higher specificity for HCC treatment is urgently warranted.

We previously reported that deregulation of epigenetic regulators is a common feature of human HCC. Meanwhile, the pathological roles of histone methyltransferase EZH2^35^, SUV39H1^36^, SETDB1^37^, and G9a^38^ as well as chromatin remodeler HELLS^39^ in liver carcinogenesis have been characterized, revealing that epigenetic aberrations are profoundly implicated in HCC development. More recently, we also reported that upregulation of the bromodomain-containing protein BRD4 contributes to aberrant superenhancer formation in HCC, and genetic ablation or pharmacological inactivation of BRD4 markedly abolished superenhancer-mediated oncogene expression and thereby suppressing HCC growth^40^. These findings highlight the critical roles of bromodomain-containing proteins in cancer development and suggest that targeting bromodomain-containing proteins is emerging as a new strategy for cancer treatment. However, apart from BRD4^41^, the roles of other bromodomain-containing proteins in liver carcinogenesis remain uncertain. In this study, we systematically analyzed the expression changes of 43 bromodomain-containing genes and identified *BRPF1* as the most frequently upregulated bromodomain-containing gene in human HCC.

BRPF1 is a component of the MOZ/MORF acetyltransferase complex, which was found to be enriched at the *HOXA9* promoter^42^. It has been reported that *MOZ/MORF* is recurrently fused to *EP300*, *CBP*, or *TIF2*, resulting in mistargeted acetylation and enhanced cancer progression in leukemia^35,43,44^. Depletion of BRPF1 reduced MOZ-TIF2 localization on *HOX* gene and lower HOX expression in acute myeloid leukemia through MOZ-dependent histone acetyltransferase activity, suggesting that BRPF1 and MOZ/MORF complex are oncogenic^23^. However, a recent report suggested *BRPF1* is a tumor suppressor gene by showing that truncated BRPF1 interacted with smoothened to promote adult SHH medulloblastoma, while the expression of BRPF1 in medulloblastoma is significantly lower compared to non-tumors^45^. The authors did not demonstrate whether re-expression of wild-type BRPF1 suppresses the carcinogenesis with BRPF1 truncation or the oncogenic role of BRPF1 that they suggested is due to the gain of function of the truncated BRPF1 protein. Moreover, the BRPF1 expression is lower in medulloblastoma, while in our study, BRPF1 expression is upregulated in several cancer types. It implies that BRPF1 may have cell-type specific function. In general, the pathological role of BRPF1 in cancer development remains largely elusive.

Our study demonstrated that BRPF1 upregulation was highly relevant to clinicopathological features. High BRPF1 expression was associated with poorer overall and disease-free survival in HCC patients. BRPF1 was also identified as a potential tissue biomarker for HCC detection. Experimentally, we showed that *BRPF1* gene ablation reduced cell proliferation rate and orthotopic xenograft tumor growth in mice. GSK5959 suppressed HCC cell growth and demonstrated therapeutic potential in vivo. More importantly, BRPF1 contributed to liver cancer stemness by increasing the expression of stem cell factors and promoting self-renewal ability. In fact, BRPF1 functions as a master regulator to expedite the expression of multiple key oncogenes involved in various cancer-promoting functions. In this study, we validated two well-known oncogenes, *E2F2* and *EZH2*, as the downstream targets of BRPF1. E2F2 is a transcription activator that regulates the expression of genes involved in cell cycle progression^46^, while EZH2 is an H3K27 methyltransferase that was reported to transcriptionally silence the genes associated with differentiation^47^. Besides, we have previously demonstrated that EZH2 inhibition reduced HCC development both in vitro and in vivo^35^.

MOZ/MORF complex regulates multiple histone acetylation marks, including H3K9ac, H3K14ac, and H3K23ac^48^. Our data demonstrated that the global acetylation levels at H3K9, H3K14, and H3K23 were unaltered upon BRPF1 inhibition, but intriguingly reduced upon MOZ/MORF inhibition. Moreover, WM1119 reduced MOZ chromatin binding more drastically than GSK5959. These data suggested that the MOZ/MORF chromatin binding and the regulation of histone acetylation by MOZ/MORF are partially BRPF1-independent. It has been reported that MOZ/MORF interacts with various transcriptional factors, such as RUNX^49^ and NRF2^50^, to regulate transcriptional activation in carcinogenesis. Interestingly, *E2F2* promoter region has a RUNX1 binding site. Whether RUNX1 or other transcriptional factors contribute to MOZ/MORF complex-independent function of MOZ/MORF or help determine the genomic localization of MOZ/MORF complex remains to be explored. Our data also showed that MOZ/MORF work with BRPF1 to increase the expression of *E2F2* and *EZH2* by promoting the H3K14 acetylation but not H3K9 or H3K23 acetylation at their promoter regions. It has been reported that MOZ/MORF complex regulated specific histone mark in different genes and cell types. For example, MOZ regulated *HOX* gene expression through H3K9 acetylation but not H3K14 acetylation in mouse embryo^17^. Another study showed that only H3K23 acylation was deregulated in neurodevelopmental disorder with *BRPF1* mutation, while H3K9 and H3K14 acetylation levels were unaltered^51^. The current research finding suggests that the regulation of MOZ/MORF complex on histone acetylation is cell-type specific and gene-specific. How MOZ/MORF complex specifically regulates its target genes through specific histone acetylation requires further investigation. Our study revealed the critical roles of BRPF1 and MOZ/MORF acetyltransferase complex as well as the specific regulation of MOZ/MORF complex on the histone acetylation mark on the promoters of its target genes in liver carcinogenesis. Our result suggested that BRPF1 is a potential target for cancer epigenetic therapy.

Bromodomain-containing proteins have gained increased attention owing to the impressive anticancer effect of BET inhibitors in preclinical studies. The BET family is a subfamily of bromodomain-containing proteins. The BET family consists of four members, namely BRD2, BRD3, BRD4, and BRDT, each of which contains two N-terminal bromodomains (BD1 and BD2) and an extra-C terminal (ET) domain. BET inhibitors target the two N-terminal bromodomains of BET proteins with a slight preference for either domain. JQ1 is the first potent and well-studied BET inhibitor. It was reported that JQ1 competitively bound to the bromodomain of BRD4 and showed great antitumor efficacy in NUT midline carcinoma (NMC) with recurrent oncogenic translocation product, BRD4-NUT^52^. This finding is further supported by a drug screening in NMC cell lines^53^. JQ1 exerts anticancer effects by regulating gene transcription. It was reported that JQ1 depleted enhancer-bound BRD4 and hence suppressed BRD4-dependent transcription, such as c-Myc^54^ and PD-L1^55^. Further study revealed that cotreatment of JQ1 and anti-PD-L1 antibody synergistically suppressed *MYC*-driven lymphoma in mice^56^, indicating that JQ1 is a potential adjuvant that could enhance the efficacy of immune checkpoint inhibitors. In fact, many reports have demonstrated that there is synergistic inhibitory effect of different drug combinations using BET inhibitors in multiple cancer types, for example, epigenetic inhibitors^57^, cell cycle inhibitors^58^, DNA damaging repair inhibitors^59^, and chemotherapeutic agents^60^, implying that targeting bromodomain-containing proteins by inhibitors has potential clinical benefits in cancer treatment. A recent study on the epigenetic landscape of HCC also showed that BET inhibitor JQ1 reduced tumor burden in a HCC mouse model^61^. Currently, many BET inhibitors with promising therapeutic potential are being developed and tested in clinical trials for the treatment of multiple cancers and metabolic diseases. Although the results from these clinical trials are still largely unavailable now, some encouraging preliminary results, such as the anticancer effect of two BET inhibitors called OTX015^62,63^ and ABBV-075^64^, have been reported. Yet, the roles of other bromodomain-containing proteins in cancer progression and the therapeutic effects of their specific inhibitors are still largely unknown. In this study, we used GSK5959, a BRPF1-specific inhibitor, and pan-BRPF inhibitors OF-1 and NI-57 for supplementary evidence to demonstrate that pharmacological inactivation of BRPF1 induced cell cycle arrest and cellular senescence, inhibited liver cancer stemness in vitro and significantly suppressed HCC tumor growth in vivo. Our findings not only demonstrated the therapeutic value of BRPF1 inhibitor in HCC treatment but also highlighted the emerging concept of targeting bromodomain-containing proteins as a new strategy for cancer treatment.

## Methods

### Clinical samples, cell lines, and small molecule inhibitors

Primary HCC samples and the corresponding non-tumor tissues were collected from HCC patients during liver resection at Queen Mary’s Hospital, Hong Kong. The use of clinical specimens was approved by the institutional review board of the University of Hong Kong and the Hong Kong Hospital Authority. Hep3B, PLC/PRF/5, HEK293FT, and HEK293T were purchased from American Type Culture Collection (ATCC). MHCC97L was provided by Dr. Z. Y. Yang from Fudan University, Shanghai, while Huh-7 was obtained from Dr. H Nakabayashi from Hokkaido University, Japan.

The small molecule inhibitors used in this study were GSK5959 (Cayman Chemical and MedKoo Biosciences), OF-1 (Cayman Chemical), NI-57 (Cayman Chemical), mithramycin A (Sigma Aldrich), WM1119 (Tocris Bioscience), and EPZ6438 (Cayman Chemical).

### Mice

4–6-week-old male BALB/c nude mice were used in the subcutaneous injection model, while 6–8-week-old male BALB/c nude mice were used in the orthotopic xenograft model. Mice were obtained and maintained in Laboratory Animal Unit, HKU throughout the experiment.

### Transcriptome sequencing

The global gene expression of 16 pairs of HCC samples and non-tumor samples was detected by RNA sequencing (RNA-seq). The sequencing reads were aligned against the human reference genome, hg19 by RNA-seq specific aligner, tophat2. Then, the isoform expression in the unit of fragments per kilobase of transcripts per million mapped reads (FPKM) of each gene was calculated by cufflinks with default parameters. The expression of bromodomain-containing genes was visualized in a heatmap. The global gene expression of HCC cell lines treated with GSK5959, WM1119, or EPZ6438 was also examined by RNA-seq. The RNA-seq data was normalized to non-tumor samples or mock control samples treated with DMSO. The RNA-seq raw data can be accessed through Bioproject (Accession ID: PRJNA701710, PRJNA701712, PRJNA701713, and PRJNA701714).

### *BRPF1* knockout by CRISPR/Cas9 genome editing system

*BRPF1* knockout cell line was established with the CRISPR/Cas9 system. MHCC97L cells carrying Cas9 were transfected with single guide RNA targeting *BRPF1* (sgBRPF1) or a non-target control (sgNTC) through a lentiviral method. The transfected cells were selected by puromycin. Successful knockout of *BRPF1* was confirmed by Sanger sequencing and tide analysis.

### Gene knockdown by shRNA or siRNA

BRPF1 knockdown cell lines were established by transfecting shRNA targeting BRPF1 (shBRPF1#1 and shBRPF1#2) into MHCC97L. MOZ, MORF and BRPF1 expression was suppressed by introducing a respective gene-specific siRNA into MHCC97L by using Lipofectamine 3000 (Life Technologies). The knockdown efficiency was determined by qRT-PCR.

### BRPF1 overexpression by CRISPR/dCas9-SAM activation system

Two sgRNAs targeting *BRPF1* promoter were transfected into MHCC97L expressing dCas9-VP64 and MS2-p65-HSF1. The overexpression efficiency was determined by qRT-PCR.

### In vitro functional assays

For the sphere formation assay, 200 Huh-7 cells in tumorsphere medium were seeded onto a single well of a 96-well plate. Twenty wells were seeded for each treatment group. The cells were allowed to grow for a week and then counted under a microscope. The β-galactosidase assay was performed by using Senescence β-Galactosidase Staining Kit (Cell Signaling) according to the supplier’s instruction.

### Flow cytometry

The cell cycle profile was detected by flow cytometry (BD Biosciences) after staining cells with propidium iodide (Calbiochem). Apoptosis assay was performed by using Annexin V-FITC Apoptosis Detection Kit (Vazyme Biotech).

### Orthotopic xenograft model

A total of 2 × 10^6^ HCC cells (sgNTC and sgBRPF1 with luciferase reporter) were resuspended in 25 μl of Matrigel and Dulbecco’s modified Eagle’s medium, high glucose (DMEM-HG) in a 1:1 ratio and injected into the left lobe of BALB/c nude mice at the age of 6–8 weeks. After 5 weeks, liver tumor formation and lung metastasis in the mice were examined by IVIS 100 Imaging System (Xenogen).

### Subcutaneous injection and in vivo drug treatment

MHCC97L cells (2 × 10^6^) were resuspended in 100 μl of Matrigel and DMEM-HG at a 1:1 ratio and injected into the right dorsal side of BALB/c nude mice at the age of 4–6 weeks. Then, the mice were treated with either mock (DMSO, Sigma Aldrich) or GSK5959 (30 mg/kg) via intraperitoneal injection once per day for 2 weeks.

### Western blotting

Whole cell lysates from HCC cells were extracted with NETN buffer. Histone proteins were extracted using an acid–base histone extraction protocol. The antibodies used were as follows: anti-EZH2 (1:1000, Cell Signaling Technology, #5246), anti-E2F2 (1:1000, Santa Cruz, sc-9967), anti-α-tubulin (1:1000, Cell Signaling Technology, #2148), anti-H3K9ac (1:1000, Cell Signaling Technology, #9649 S), anti-H3K14ac (1:1000, Cell Signaling Technology, #7627 S), anti-H3K23ac (1:1000, Millipore, 07-355), and anti-histone H3 (1:1000, Millipore, 05-928).

### Chromatin immunoprecipitation assay

H3K9ac, H3K14ac, H3K23ac, and histone H3 chromatin immunoprecipitation assays were performed using EZ-Magna ChIP™ HiSens Chromatin Immunoprecipitation Kit (Merck Millipore) according to the manufacturer’s instruction. The eluted fragments were purified by a PCR purification kit. Normal rabbit IgG (Millipore, 12-370), normal mouse IgG (Millipore, 12-371), MOZ (Santa Cruz, sc-293283), anti-H3K9ac (Cell Signaling Technology, #9649S), anti-H3K14ac (Cell Signaling Technology, #7627S), anti-H3K23ac (Millipore, 07-355), and anti-histone H3 (Millipore, 05-928) were used to precipitate the fragments attached to the target histones. The immunoprecipitation of the targeted region was then determined by qRT-PCR and calculated using percentage input method.

### Statistics and reproducibility

Gene expression levels between HCC samples and non-tumorous liver samples were compared by paired *t*-test. The tumor volumes and tumor weights between the control subcutaneous tumors and the tumors in sgBRPF1 group were also compared by *t*-test. The Mann–Whitney *U* test was used to analyze continuous nonparametric data, while the independent *t*-test was used to analyze continuous parametric data. Survival rate analysis was performed by the Kaplan–Meier method and log-rank test. Linear regression was used to test the correlation between two gene expression. Correlations of categorical data was determined by Chi-Square test. Statistical analyses were carried out by Prism 8 software. Error bars indicate mean ± SD. Results were repeated at least three times unless indicated otherwise. The HKU HCC cohort contains 16 pairs of non-tumorous liver and HCC tumors, while TCGA database on HCC contains 50 non-tumors and 371 tumors. TCGA database on colorectal and kidney cancer contains 32 pairs of non-tumor and tumor samples respectively.

### Supplementary information

Detailed methodology can be found in the Supplementary Methods. Further details about sgRNA sequences, sequencing primer sequences, qRT-PCR primer sequences, ChIP primer sequences and cloning primer sequences can be found in the Supplementary Table.

### Reporting summary

Further information on research design is available in the Nature Research Reporting Summary linked to this article.

## Supplementary information

Supplementary Information

Description of Additional Supplementary Files

Supplementary Data 1

Reporting Summary

## Data Availability

RNA-seq data from this study are available in NCBI BioProject (Accession ID: PRJNA701710, PRJNA701712, PRJNA70173, and PRJNA701714). The source data including uncropped blots underlying Figs. 1–9 and Supplementary Fig. 1-10 are provided as a Supplementary Data file. Publicly released microarray data are available via GEO (accession GSE23450 and GSE23451). RNA-seq data from in-house clinical samples can be accessed through NCBI Bioproject (Accession ID: 294031). A reporting summary for this article is available as a supplementary information file. Any remaining information can be obtained from the corresponding author upon reasonable request.
