# Supplementary material for: Bromodomain-containing protein BRPF1 is a therapeutic target for liver cancer
Source: Commun Biol. 2021 Jul 20;4:888. doi: 10.1038/s42003-021-02405-6 (PMC8292510; doi:10.1038/s42003-021-02405-6)
Supplement: Supplementary file 1 — Supplementary Information [file 42003_2021_2405_MOESM1_ESM.pdf]

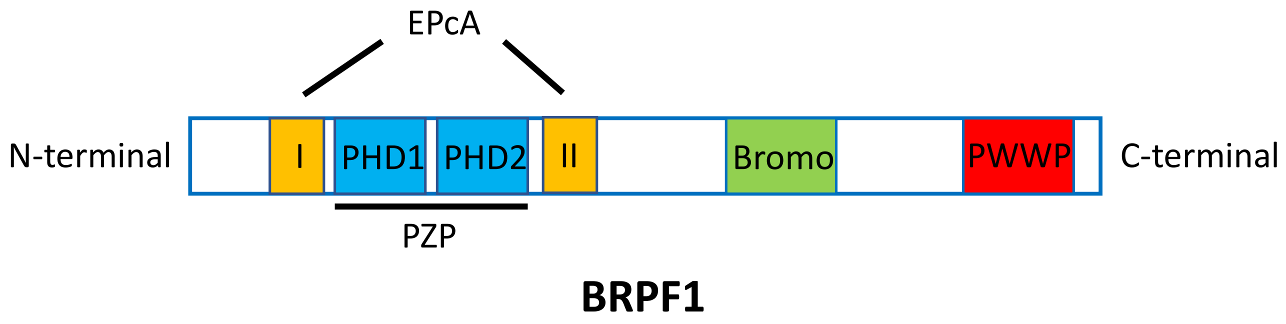


**Supplementary Figure S1.** A schematic representation of human BRPF1 protein. BRPF1 contains a double PHD and zinc finger module (PZP), a bromodomain and a C-terminal PWWP domain for recognition of various histone marks. The function of enhancer of polycomb-like motif (EPcA) is poorly defined.

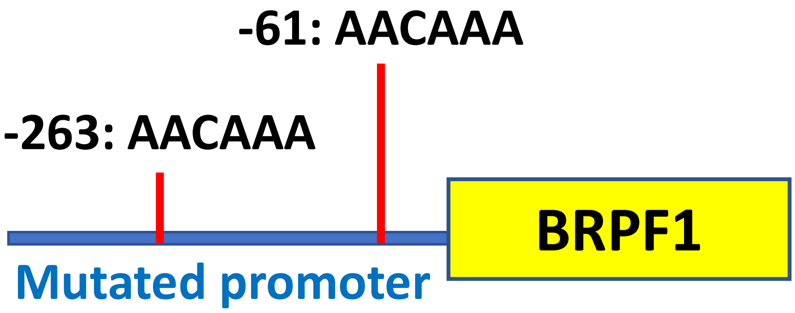


**Supplementary Figure S2.** BRPF1 promoter was mutated by changing guanines in SP1 binding sites to adenines.

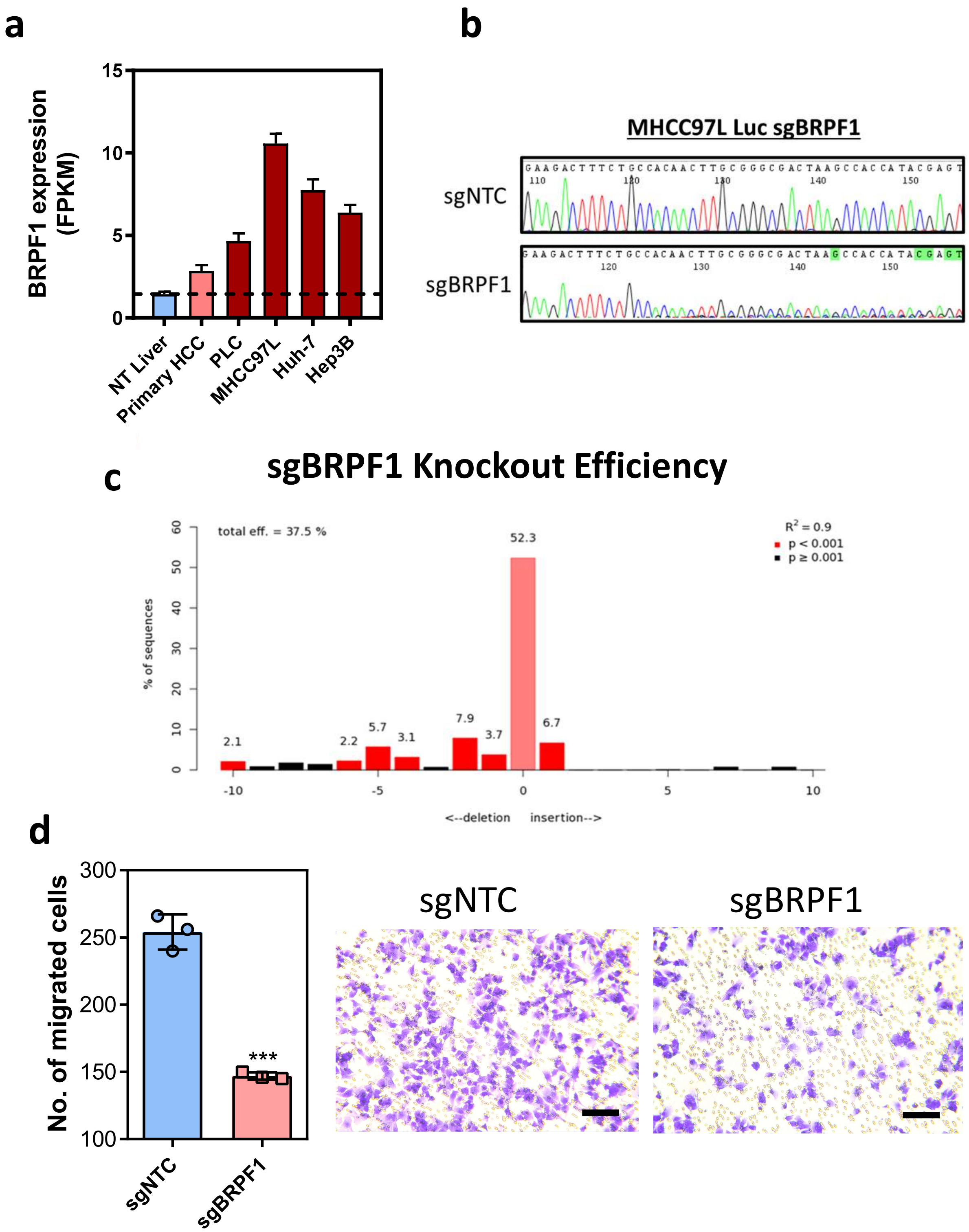


**Supplementary Figure S3. a** Expression of BRPF1 in HCC cell lines determined by RNA-Seq. **b** Knockout efficiency of BRPF1 (sgBRPF1) in MHCC97L validated by PCR and subsequent Sanger sequencing. **c** Tide analysis demonstrating that the CRISPR knockout successfully induced indels in MHCC97L. **d** BRPF1 knockout suppressed cell migration in MHCC97L (Scale bar: 0.3 mm). Error bars indicate mean ± SD. Data were compared by independent *t*-test. Results were repeated at least three times. ***P<0.001 vs. sgNTC as indicated.

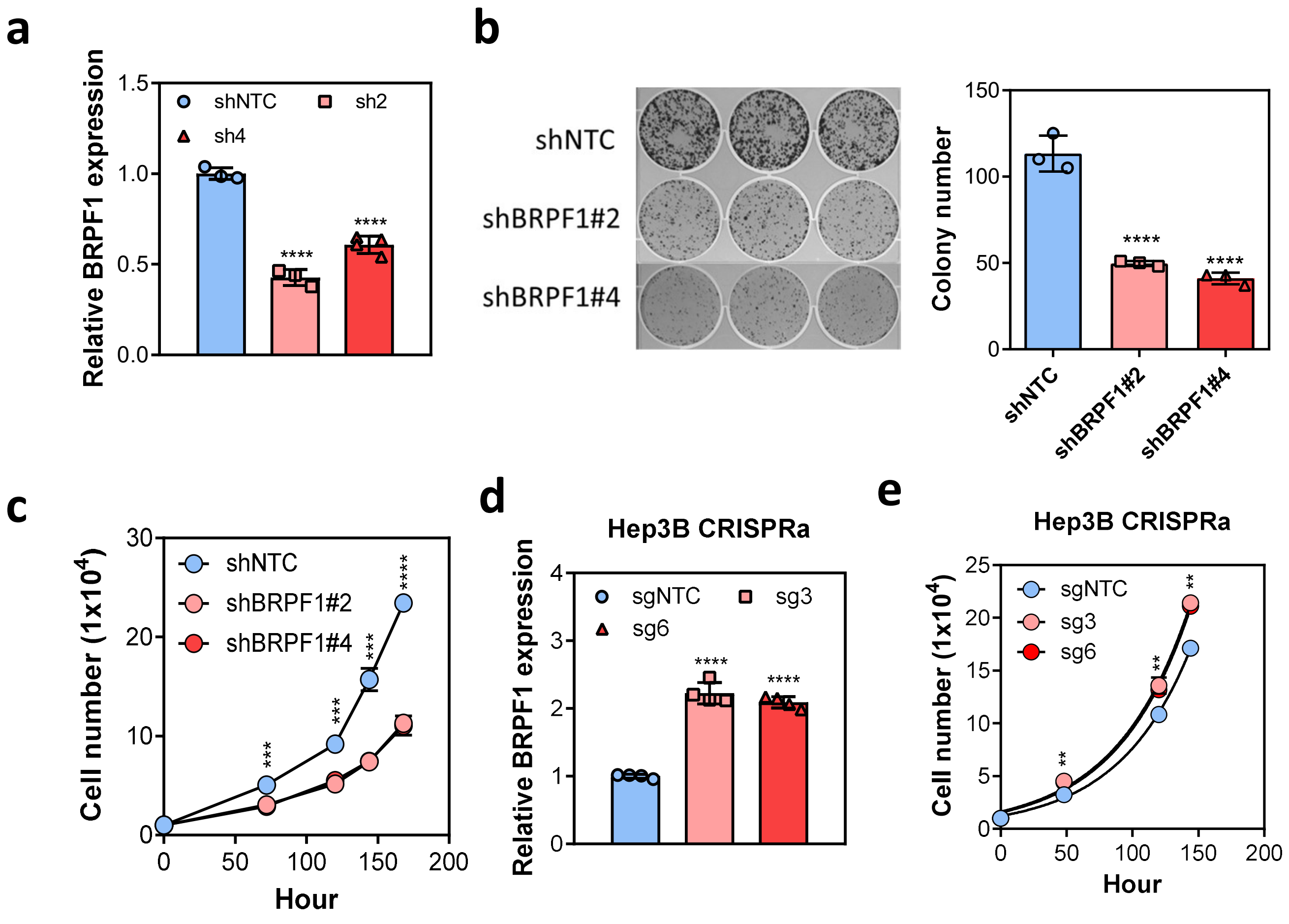


**Supplementary Figure S4. a** Knockdown efficiency of BRPF1 in MHCC97L cells upon shRNA-mediated knockdown. **b** BRPF1 knockdown decreased cell colony formation in MHCC97L. **c** BRPF1 knockdown decreased cell proliferation in MHCC97L. **d** Overexpression efficiency of BRPF1 in Hep3B upon CRISPR activation. **e** BRPF1 upregulation increased cell proliferation in Hep3B. **P<0.01, ***P<0.001, ****P<0.0001 vs. shNTC or sgNTC as indicated.

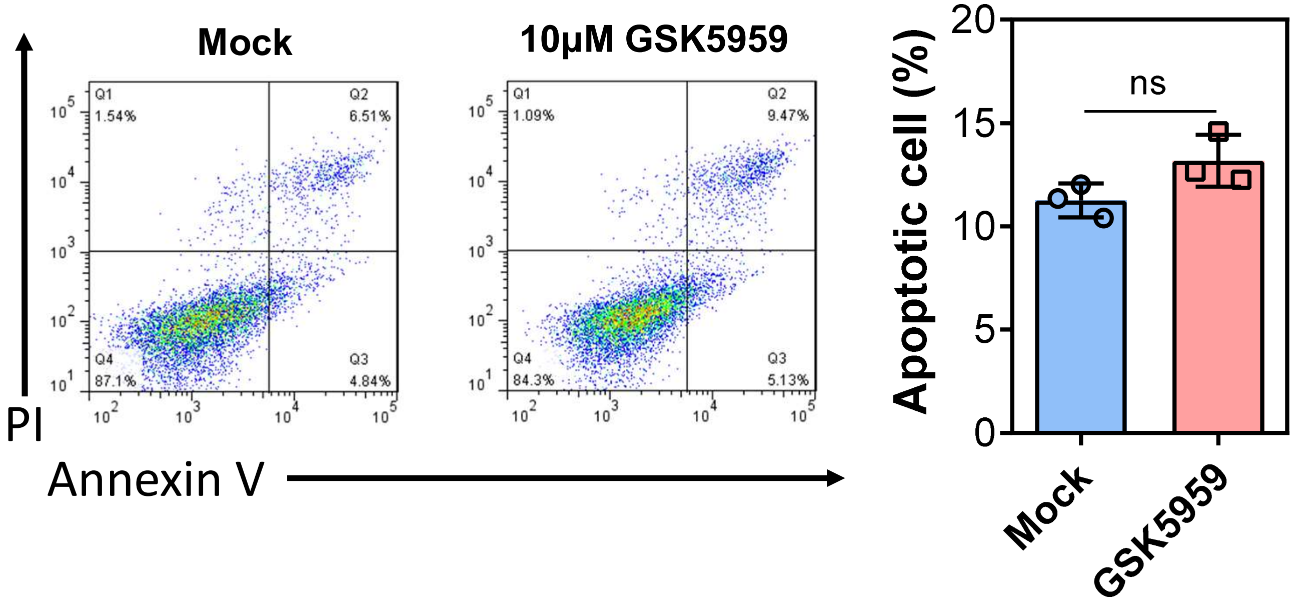


**Supplementary Figure S5.** GSK5959 treatment for 48 hours had no significant effect on apoptosis in MHCC97L. Error bars indicate mean ± SD. Data were compared by independent *t*-test. Results were repeated at least three times. ns: not significant vs. mock as indicated.

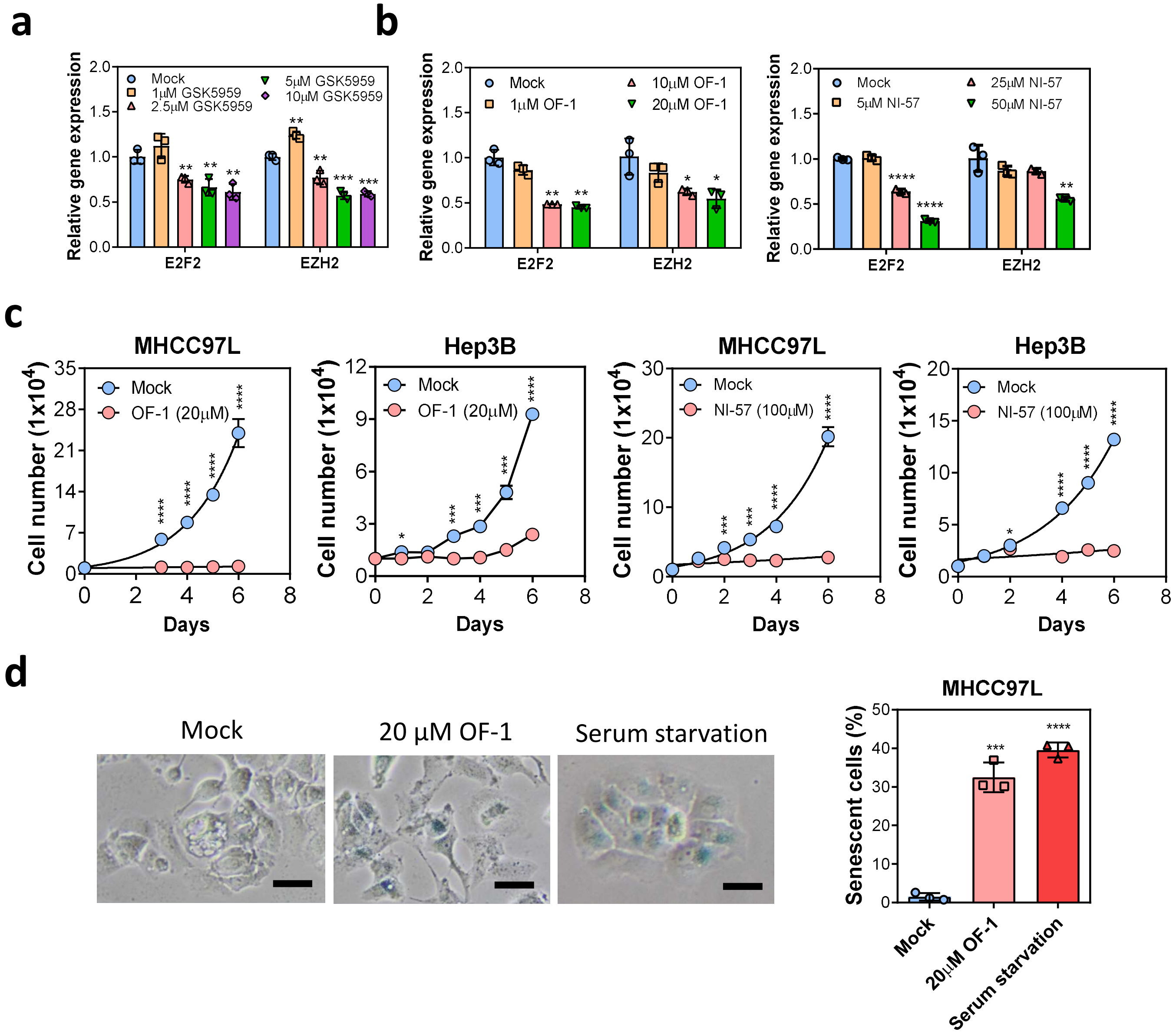


**Supplementary Figure S6. a, b** The mRNA expression of E2F2 and EZH2 was reduced by GSK5959, OF-1 and NI-57 in a dose-dependent manner after 5-day treatment. **c** The cell proliferation rate was reduced upon 20 µM OF-1 or 100 µM NI-57 treatment in MHCC97L and Hep3B cells. **d** 20 µM OF-1 significantly induced cellular senescence after 5-day treatment (Scale bar: 0.3 mm). Serum-starved cells acted as a positive control. Error bars indicate mean ± SD. Data were compared by independent *t*-test. Results were repeated at least three times. *P<0.05, **P<0.01, ***P<0.001, ****P<0.0001 vs. mock as indicated.

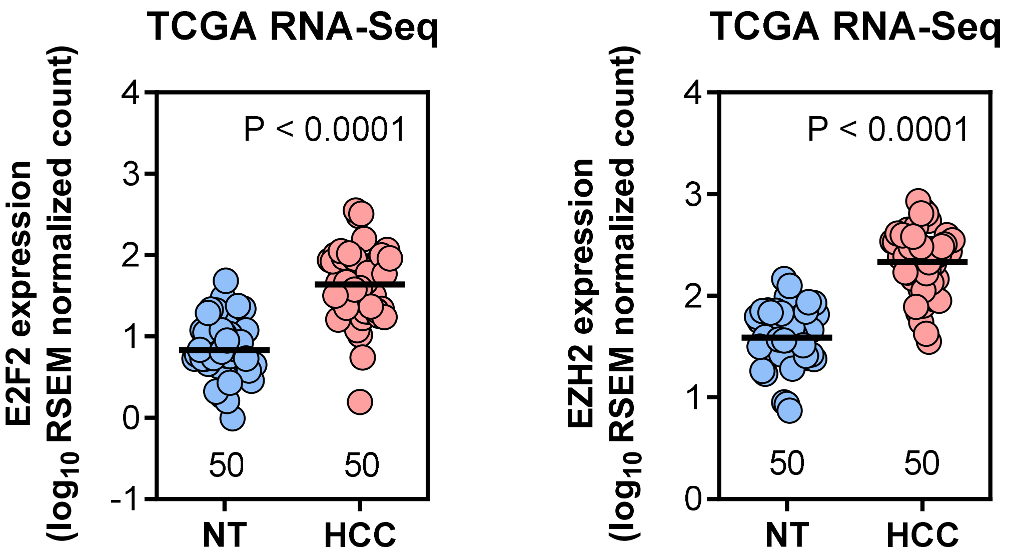


**Supplementary Figure S7.** E2F2 and EZH2 were highly upregulated in liver cancer (TCGA data, paired *t*-test). The numbers shown in the graphs represent the number of samples in each group.

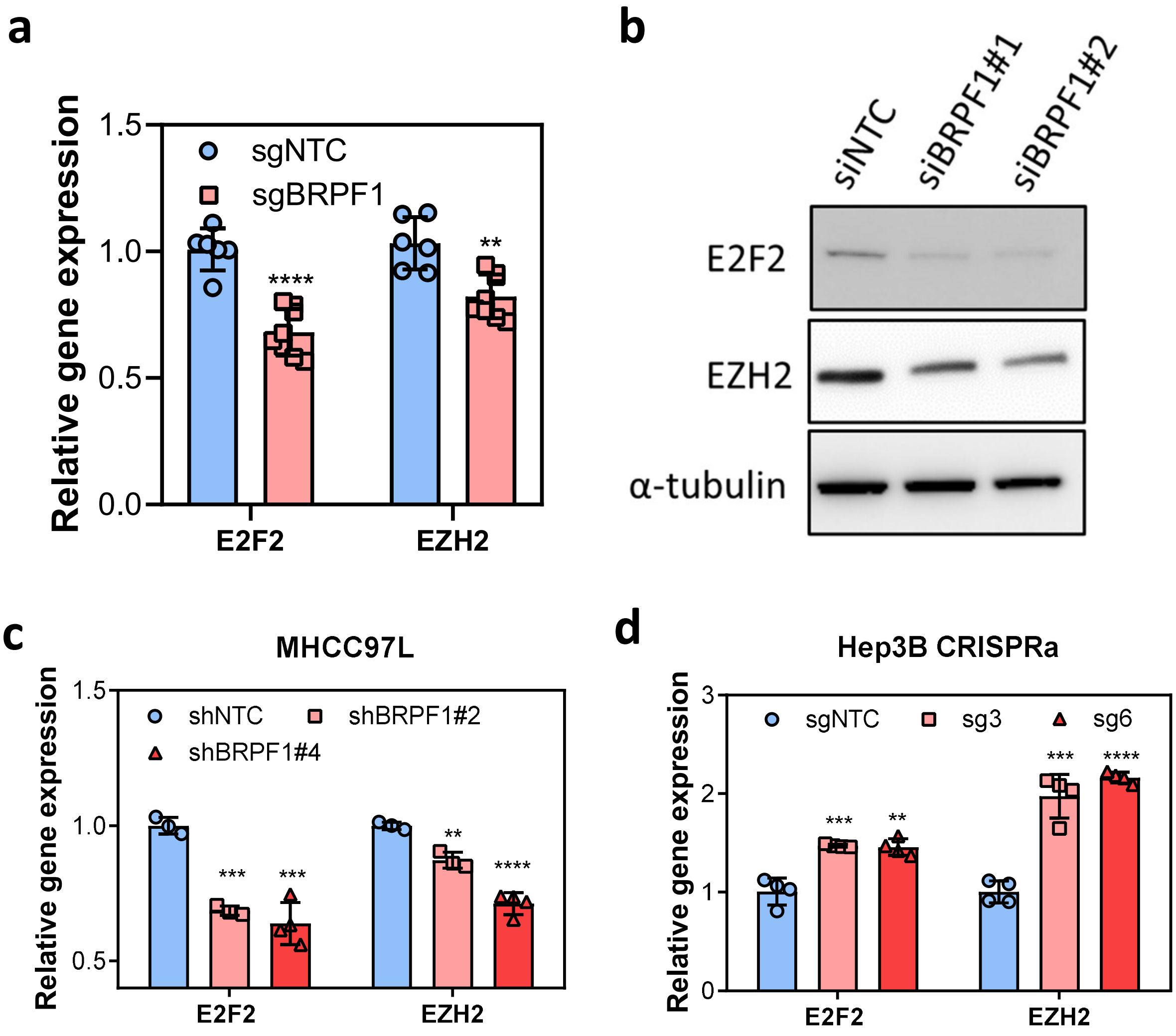


**Supplementary Figure S8. a** The expression of E2F2 and EZH2 was significantly reduced in the orthotopic liver tumors with sgBRPF1 cells, compared to the tumors with sgNTC cells. **b** BRPF1 knockdown by gene-specific siRNA reduced the protein expression of E2F2 and EZH2 in MHCC97L. **c** BRPF1 knockdown reduced the expression of E2F2 and EZH2 in MHCC97L. **d** BRPF1 overexpression upregulated the expression of E2F2 and EZH2 in Hep3B. Error bars indicate mean ± SD. Data were compared by independent *t*-test. Results were repeated at least three times. $^{**}P<0.01$, $^{***}P<0.001$, $^{****}P<0.0001$ vs. sgNTC or shNTC as indicated.

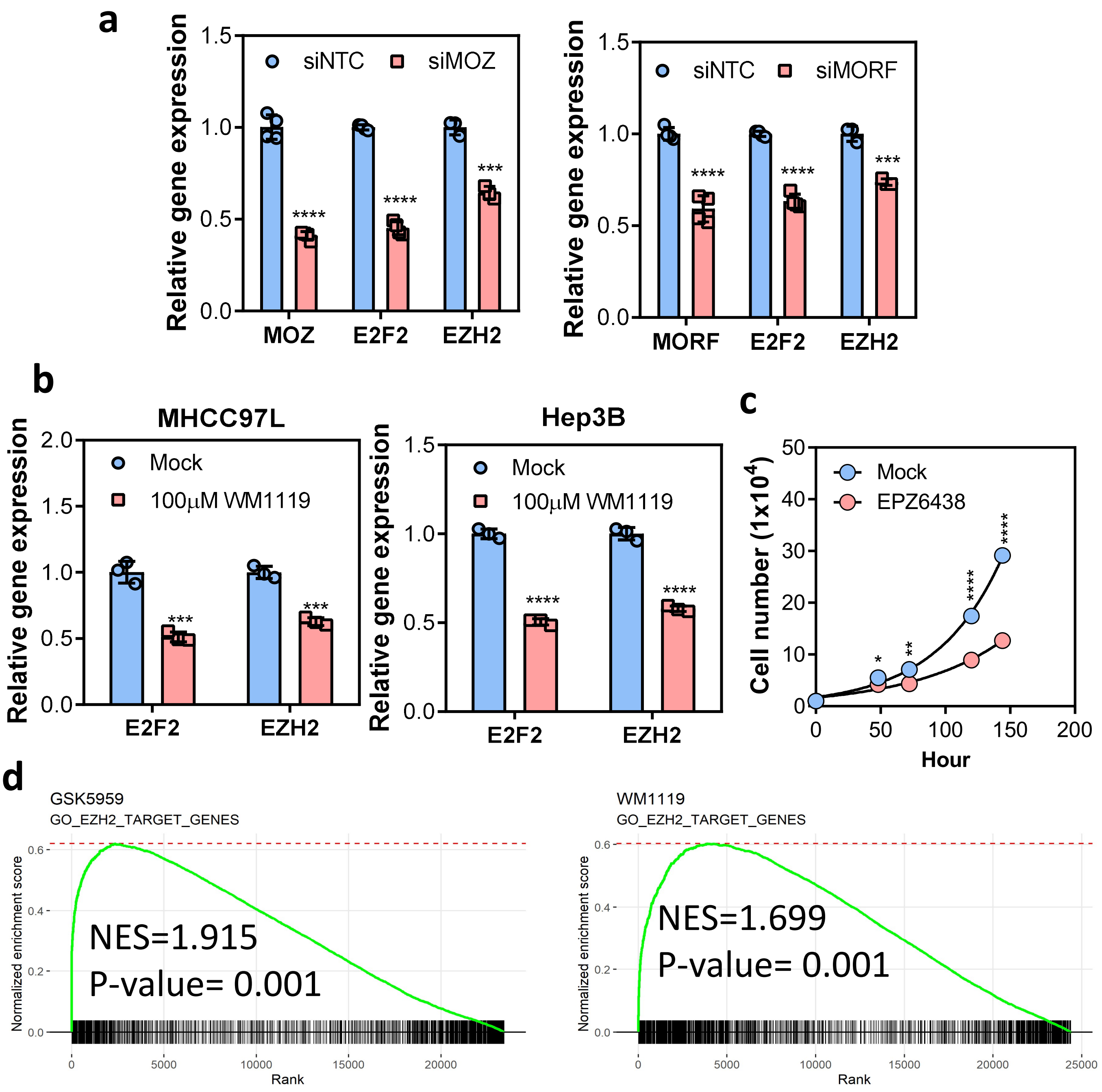


**Supplementary Figure S9. a** siRNA-mediated depletion of MOZ or MORF reduced the expression of E2F2 and EZH2 in MHCC97L cells after 72-hour transfection. **b** The mRNA expression of E2F2 and EZH2 was downregulated by 100 μM WM1119 in MHCC97L and Hep3B after 72-hour treatment. **c** 50 μM EPZ6438 significantly decreased cell proliferation in MHCC97L**. d** GSEA results showed that differentially expressed genes in GSK5959 or WM1119 treated MHCC97L were enriched for EZH2 target genes (Genes with fold change>2 upon EPZ6438). Error bars indicate mean ± SD. Data were compared by independent *t*-test. Results were repeated at least three times. *P<0.05, **P<0.01, ***P<0.001, ****P<0.0001, ns: not significant vs. siNTC or mock as indicated.

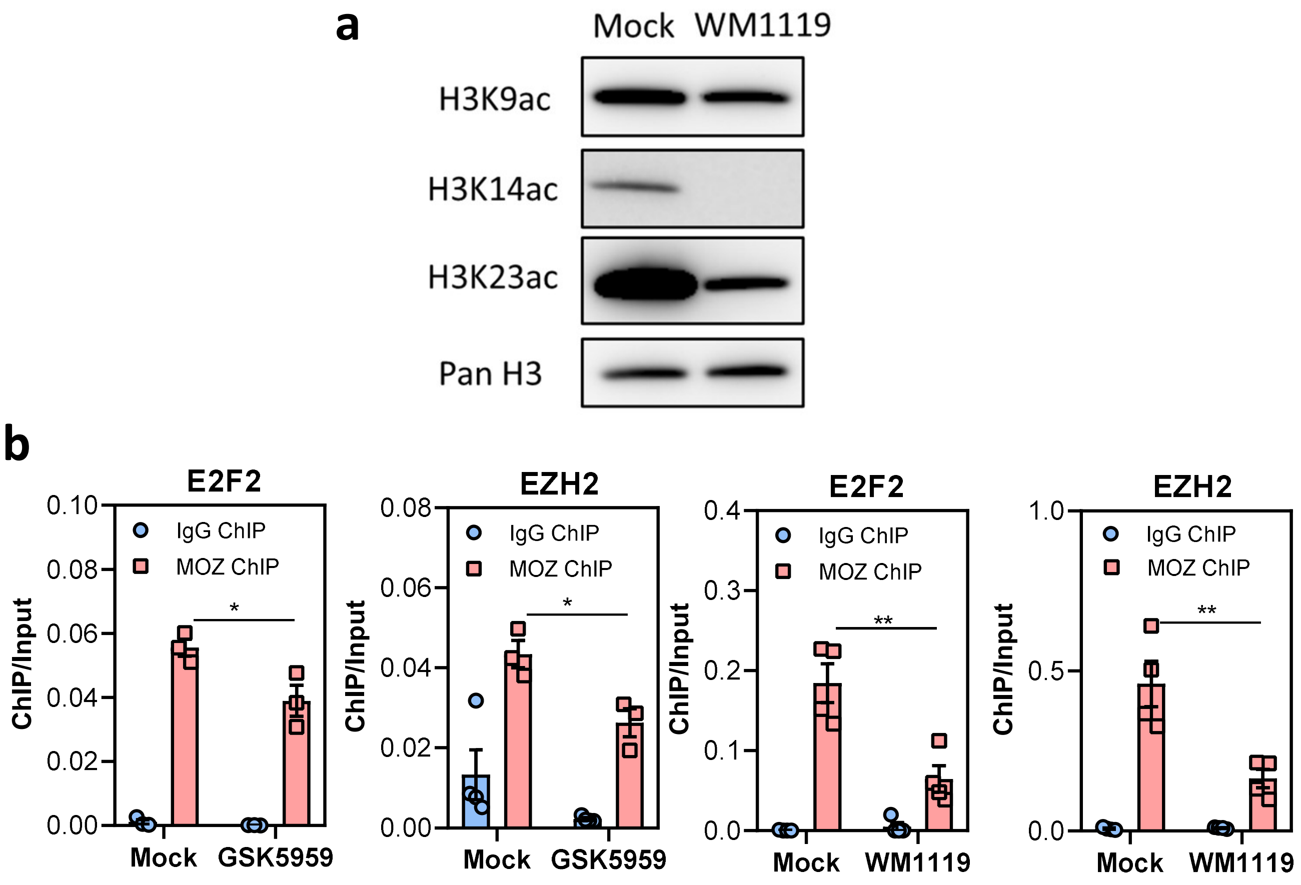


**Supplementary Figure S10. a** The global acetylation levels at H3K9, H3K14 and H3K23ac were reduced upon 100 µM WM1119 treatment for 72 hours. **b** MOZ genomic localization levels on E2F2 and EZH2 promoters were reduced upon 10 µM GSK5959 for 48 hours or 100 µM WM1119 for 72 hours in MHCC97L. Error bars indicate mean ± SD. Data were compared by independent *t*-test. Results were repeated at least three times. *P<0.05, **P<0.01, ns: not significant vs. IgG ChIP as indicated.

# Supplementary Methods

## Clinical Samples

A total of 16 HCC Chinese patients with HBV chronic infection were involved in this study. The informed consent was obtained from the patients. Primary HCC samples and the corresponding non-tumor samples were collected from HCC liver resection at Queen Mary's Hospital, Hong Kong. The clinical samples collected were either snap-frozen in liquid nitrogen and kept at -80°C or fixed in formalin and embedded in paraffin. The use of clinical specimens was approved by the institutional review board of the University of Hong Kong and the Hong Kong Hospital Authority. The RNA-sequencing data on the 16 paired HCC and nontumor samples can be accessed through Bioproject (Accession ID: 294031).

## TCGA Dataset

The RNA-sequencing data on 50 pairs of HCC and non-tumor samples was retrieved from The Cancer Genome Atlas (TCGA) through broad institute (http://gdac.broadinstitute.org/). The clinical data from the HCC cohort (TCGA, PanCancer Atlas, 372 samples) was obtained through cBioportal (https://www.cbioportal.org/). 315 TCGA HCC samples were analyzed for BRPF1 gene copy number.

## Microarray Data

Publicly released microarray data are available via GEO (accession GSE23450 and GSE23451). The microarray data showed the BRPF1 expression in CD133+ HCC cell population and CD133- counterpart. The BRPF1 expression in CD133+ group was normalized to that in CD133- group.

## Cell Lines

Human hepatoma cell lines or simply HCC cell lines including Hep3B, PLC/PRF/5, human embryonic kidney cell line HEK293FT and HEK293T were purchased from American Type Culture Collection (ATCC). HCC cell line MHCC97L was given by Dr. Z. Y. Yang from Fudan University, Shanghai, while Huh-7 was obtained from Dr. H Nakabayashi from Hokkaido University Scholl of Medicine, Japan. Cell line authentication test has been performed by using AuthentiFiler™ PCR Amplification Kit (Applied Biosystems). Hep3B cells were cultured in Minimum Essential Media (MEM, Life Technologies) with 1 mM sodium pyruvate. PLC/PRF/5 cells were cultured in MEM. Huh-7, human embryonic kidney cell line HEK293FT and HEK293T were cultured in Dulbecco's Modified Eagle Medium high glucose (DMEM-HG, Life Technologies). MHCC97L cells were cultured in DMEM-HG with 1 mM sodium pyruvate. Culture medium was supplemented with 10% fetal bovine serum (Life Technologies) and 50 U/ml penicillin-streptomycin (Life Technologies).

**Mice**

Male BALB/c nude mice were used in all animal experiments. Mice at the age of 4 to 6 weeks were used in the subcutaneous injection model and *in vivo* drug treatment, while mice at the age of 6 to 8 weeks were used in the orthotopic xenograft model. Mice were obtained and maintained in Laboratory Animal Unit, HKU throughout the experiment. Animal experiments were performed with the approval of Committee on the Use of Live Animals in Teaching and Research (CULATR) of the University of Hong Kong. All experimental procedures followed strictly to the animals (Control of Experiments) ordinance of Hong Kong.

**Small Molecule Inhibitors**

GSK5959 was purchased from Cayman Chemical and Medkoo Biosciences. OF-1, NI-57 and EPZ6438 were bought from Cayman Chemical. WM1119 was bought from Tocris Biosciences. Mithramycin A was purchased from Sigma Aldrich.

**Transcriptome Sequencing and Computational Analyses**

The global gene expression profiles of mock control cells and HCC cells treated with GSK5959, WM1119 or EPZ6438 were examined by transcriptome sequencing (RNA-seq). The RNA samples were sent for sequencing using Illumina NovaSeq 6000 with the read length of paired-end 150 bp. The sequencing data were aligned to hg19 using Hisat2, while the expression level in fragments per kilobase of transcript per million mapped reads (FPKM) was called by Stringtie. The expression of bromodomain-containing genes was visualized in a heatmap. The RNA-sequencing raw data can be found in Bioproject (Accession ID: PRJNA701710, PRJNA701712, PRJNA701713 and PRJNA701714). Pathway analysis on the common downregulated genes upon GSK5959 treatment in HCC cells was performed by DAVID functional annotation tool, while the gene set enrichment analysis was performed by R.

**Luciferase Reporter Assay**

To determine *BRPF1* promoter activity, the promoter region from -1000bp to +250bp of *BRPF1* was cloned by PCR and ligated into pGL3-Basic luciferase reporter vector (Promega). Mutated *BRPF1* promoter was constructed by changing two SP1 sites GGCGGG into AACAAA through molecular cloning and then ligated into the reporter vector as well. After seeding HEK293T, 20 nM siSP1 (siSP1#1, siSP1#2) (Integrated DNA technology) or 20 nM siNTC (Sigma Aldrich) was first transfected into the cells using Lipofectamine 3000 (Life Technologies). The reporter vectors containing the *BRPF1* promoter or the mutated one were then co-transfected with pGK Renilla

luciferase control vector using Lipofectamine 3000 into HEK293T on the next day. The Firefly and Renilla luciferase activity were quantified by Dual-Luciferase Assay Kit (Promega) 48 hours after the second transfection according to the supplier's instruction. In brief, the cells were lyzed with 1x passive lysis buffer for 15 minutes at room temperature. Then, 20 µl of cell lysate was transferred into PerkinElmer 96 flat bottom white polystyrene plate. The luciferase activity was measured by luminescence plate reader (Tecan) after the addition of 50 µl 1x LARII and 50 µl 1x Stop & Glo respectively. To test the effect of mithramycin A on *BRPF1* promoter activity, the reporter vector containing the *BRPF1* promoter or the mutated one and pGK Renilla luciferase control vector were first transfected into HEK293T. After one day of transfection, 1 µM of mithramycin A was added to treat the cells for 48 hours before subsequent measurement of luciferase activity.

***BRPF1* Knockout by CRIPSR/Cas9 Genome Editing System**

A single guide RNA (sgRNA) targeting the first exon of *BRPF1* gene was designed based on the result from http://crispor.tefor.net/ and then cloned into lentiGuide-Puro (Addgene #52963). For negative control, non-target control sequence was cloned into the same vector. The plasmids were then transfected into HEK293FT together with lentivirus packaging mix plasmids (GAG, VSVG and REV) using Lipofectamine 3000. Lentiviral particles were collected after 48 hours and used to infect MHCC97L cells containing Cas9 with the help of polybrene. After 48 hours, 2 µg/ml puromycin was applied to the infected cells for positive selection. Genomic DNA from the transformed cells was extracted and used for PCR to amplify the region around the sgRNA targeting site. The PCR product was purified and sent for sanger sequencing to determine the knockout efficiency. The sgRNA and sequencing primer sequences can be found in the Supplementary Table.

## Gene Knockdown by siRNA or shRNA

MOZ, MORF and BRPF1 expression were silenced by their respective gene-specific siRNA. siNTC, siMOZ (Santa cruz), siMORF (Santa cruz) and siBRPF1 (siBRPF1#1, siBRPF1#2) (Integrated DNA technology) were separately transfected into MHCC97L by using Lipofectamine 3000. The concentration of siMOZ and siMORF used was 100 nM, while the concentration of siBRPF1#1 and #2 used was 20 nM. For shRNA-mediated knockdown model, shRNAs targeting BRPF1 (shBRPF1#2, shBRPF1#4) were cloned into pLKO.1 puro (Addgene#8453) vector, then transfected into MHCC97L cells through lentiviral method. The successfully transfected cells were selected upon puromycin treatment.

## BRPF1 Overexpression by CRISPR/dCas9 Activation System

The BRPF1 overexpressing system was established by CRISPR/dCas9 synergistic activation mediator (SAM) system. Two sgRNAs targeting BRPF1 promoter region (sgBRPF1#3 and sgBRPF1#6) were cloned into lenti sgRNA(MS2)-zeo (Addgene #61427) and then transfected into Hep3B cells containing dCas9-VP64 and MS2-p65-HSF1. The overexpression efficiency was then determined by qRT-PCR.

## Functional Assay

For cell proliferation assay, $1x10^4$ cells were seeded into a 24-well plate in triplicate. The number of HCC cells was counted using Z1 Coulter Counter Cell and Particle Counter (Beckman Coulter) for a week. For colony formation assay, 1000 HCC cells were seeded into a 6-well plate in triplicate and allowed to grow for about 2 weeks. To visualize cell colonies, the colonies were first fixed with 100% methanol for 10 minutes and then stained with 1% crystal violet with shaking overnight. The images of cell colonies were captured and counted using ImageJ software. For cell

migration assay, 5x$10^4$ cells were resuspended in 200µl of DMEM-HG only medium and loaded to the upper chamber of the transwell (Millicell® Hanging Cell Culture Insert, Millipore). 500 µl of fresh culture medium with 10% FBS was added to the lower chamber to attract cells from the upper chamber to pass through pores in the membrane of the transwell. The culture dish was then incubated in 37°C humidified CO2 chamber for 16 hours. Migrated cells on the outer side of the upper chamber were fixed by methanol (Millipore) and stained with 1% crystal violet (Sigma-Aldrich). 3 images of the transwell membrane were randomly captured and used for cell number counting by ImageJ.

**Cell Cycle Analysis**

To perform cell cycle analysis, 2x$10^5$ cells were first seeded into a 6-well plate. After 2 days, the cells were trypsinized and washed with 1x PBS. Then the cells were fixed with 70% ethanol for 4 hours. The fixed cells were treated with Rnase A for 5 minutes to degrade RNA and prevent it from interfering with the result. The cells were then stained with a fluorescent DNA-intercalating agent, propidium iodide (Calbiochem) for 20 minutes. The cell cycle profile was examined by flow cytometry (BD Biosciences) and analyzed by FlowJo software.

**Apoptosis Assay**

Apoptosis assay was performed by using Annexin V-FITC Apoptosis Detection Kit (Vazyme Biotech). Cells together with the culture medium were collected and centrifuged at 250 x g for 5 minutes at room temperature. The cells were then washed with 1x PBS twice and resuspended in 100 µl of binding buffer. 5 µl of Annexin V FITC and 5 µl of PI were added to the resuspended cells for 15-minute incubation in the dark at room temperature. Finally, 400 µl of binding buffer was added to each sample before the samples were analyzed by BD FACS flow cytometer. The cell gating and analysis were done by FlowJo 7.6.

**Sphere Formation Assay**

The number of viable cells was first counted with trypan blue. 4000 live cells were then suspended in tumorsphere medium (Dulbecco's Modified Eagle Medium/F12 with 20 ng/ml epidermal growth factor, 10 ng/ml basic fibroblast growth factor, 5 μg/ml insulin and 0.4% bovine serum albumin). 200 cells in each well were seeded for each treatment group in a 96-well plate. There were 20 replicates for each experimental group. The cells were then incubated for a week without any disturbance. The number of tumorsphere formed was counted under a phase-contrast microscope using 40X magnification lens.

**Orthotopic Xenograft Model**

$2x10^6$ HCC cells (MHCC97L sgNTC and sgBRPF1 with a luciferase gene) were resuspended in 25 μl with equal volume of Matrigel (Corning) and DMEM-HG and then injected into nude mice at the age of 6 to 8 weeks. After 5 weeks, tumor formation in the liver of mice was examined by IVIS Spectrum (Xenogen). Extrahepatic metastasis to lung was detected by *ex vivo* bioluminescent imaging. Mice livers and lungs were dissected and snap-frozen for future use.

**Subcutaneous Injection Model and *In Vivo* Drug Treatment**

$2x10^6$ MHCC97L cells were resuspended in 100 μl with equal volume of Matrigel and DMEM-HG mixture and then injected into the right dorsal side of nude mice at the age of 4 to 6 weeks. After 2 weeks, the mice were divided into 2 groups, treated with mock (DMSO, Sigma Aldrich) or GSK5959 (30 mg/kg) via intraperitoneal injection once per day for 2 weeks. The tumor size (length x $width^2$) and the weight of mice were measured every day. After 2-week treatment, the mice were sacrificed to collect tumors. Tumor weights were measured.

**Tissue Homogenization**

The mice liver tumors extracted from the orthotopic xenograft experiment were homogenized for RNA extraction. In brief, around 0.2 g of liver tissue was cut into smaller fractions by a blade and then transferred into a 2 ml tube with a screw cap and biomedical beads (MP Biomedicals) inside. 1 ml of Trizol reagent was added into each tube. The liver tissues were homogenized by Minilys personal homogenizer (Bertin) at the highest speed for 30 seconds. The thoroughly homogenized liver tissues were then proceeded to RNA extraction.

**RNA Extraction and Quantitative Real-time Polymerase Chain Reaction (qRT-PCR)**

TRIzol reagent (Invitrogen) was used to extract total RNA. The procedures strictly followed the manufacturer's protocol. 1 μg RNA was converted to complementary DNA using PrimeScript™ RT reagent kit (Takara). qRT-PCR was performed by using SYBR® Green Real-Time PCR Master Mix (Invitrogen). HPRT1 gene acted as a housekeeping gene. All qRT-PCR primers used in this study were listed in the Supplementary Table.

**Protein Extraction and Western Blotting**

Whole cell lysates from HCC cell culture were extracted with NETN buffer, while histone proteins were extracted using an acid-base histone extraction protocol. The antibodies used were as follows: anti-EZH2 (1:1000, Cell Signaling Technology, #5246), anti-E2F2 (1:1000, Santa Cruz, sc-9967), anti-α-tubulin (1:1000, Cell Signaling Technology, #2148), anti-H3K9ac (1:1000, Cell Signaling Technology, #9649S), anti-H3K14ac (1:1000, Cell Signaling Technology, #7627S), anti-H3K23ac (1:1000, Millipore, 07-355), anti-histone H3 (1:1000, Millipore, 05-928).

**β-galactosidase Assay**

Senescence β-Galactosidase Staining Kit (Cell Signaling) was used for β-galactosidase assay. $5x10^4$ cells were first seeded into a 12-well plate. GSK5959/OF-1 was added into the cells on the

next day. The cell culture was incubated for 5 days. After treatment, the cells were then washed with 1x PBS and fixed with 1x fixative solution for 5 minutes. The cells were washed again with 1x PBS twice and incubated for 48 hours with β-galactosidase staining solution prepared based on the recipe provided in the protocol. Senescent cells were stained with blue colour. The images of the cells were captured, and the number of stained cells was counted under microscope with 20X magnification.

**Chromatin Immunoprecipitation Assay**

H3K9ac, H3K14ac, H3K23ac and histone H3 chromatin immunoprecipitation assay were performed using EZ-Magna ChIP™ HiSens Chromatin Immunoprecipitation Kit (Merck Millipore). In brief, $1x10^7$ cells treated with either GSK5959 for 48 hours or WM1119 for 72 hours were harvested. DMSO was added for the mock group. After fixation, the nuclei were extracted and lyzed. The resultant chromatins were sonicated into smaller fragments. Normal rabbit IgG (Millipore, 12-370), normal mouse IgG (Millipore, 12-371), MOZ (Santa Cruz, sc-293283), anti-H3K9ac (Cell Signaling Technology, #9649S), anti-H3K14ac (Cell Signaling Technology, #7627S), anti-H3K23ac (Millipore, 07-355) and anti-histone H3 (Millipore, 05-928) were used to precipitate the fragments attached to the target histones. 5 µg of individual antibody was used for each chromatin immunoprecipitation. The fragments were then eluted and purified by PCR purification kit. The DNA expression was then determined by qRT-PCR and calculated using percentage input method.

**Statistical Analyses**

Gene expression levels between HCC samples and nontumorous liver samples were compared by paired *t*-test. The tumor volumes and tumor weights between the control subcutaneous tumors and

the tumors in sgBRPF1 group were also compared by paired *t*-test. Mann Whitney U test was used to analyze continuous non-parametric data, while independent *t*-test was used to analyze continuous parametric data. Survival rate analysis was performed by Kaplan-Meier method and Log rank test. Linear regression or Chi-square test was used to test the correlation between two gene expression. Statistical analyses were carried out by Prism 8 software. Data were represented as mean ± standard deviation (error bars). Results were repeated at least three times unless indicated otherwise. $P < 0.05$ was considered as statistically significant. ns: not significant, * $P < 0.05$, ** $P < 0.01$, ***$P < 0.001$, ****$P < 0.0001$.

# Supplementary Table

**Table S1. sgRNA Target Sequences (KO: knockout; ACT: activation)**

| sgRNA | Sequence (5’→3’) |
|---|---|
| sgBRPF1 (KO) | GCTTAGTCGCCCGCAAGTTG |
| sgNTC (KO/ACT) | CTGAAAAAGGAAGGAGTTGA |
| sgBRPF1#3 (ACT) | GGCTCGAAAGACCGTGCTCC |
| sgBRPF1#6 (ACT) | CCGGGATTCAGAAAGGACCC |

**Table S2. shRNA Oligonucleotide Sequences**

| shRNA | Sequence (5’→3’) |
|---|---|
| shBRPF1#2 | CCGGCCGAAAGGTCTACAAGAGTTACTCGAGTAACTCTTGTAGACCTTTCGGTTTTTG |
| shBRPF1#4 | CCGGCCAATGGACTTACCAGCCAATCTCGAGATTGGCTGGTAAGTCCATTGGTTTTT |

**Table S3. qRT-PCR Primer Sequences**

| Primer | Sequence (5’→3’) |
|---|---|
| Human BRPF1 | Forward: GGCCACAGCATGGTAAGGAA<br>Reverse: ACCTTCTCGGGGCATCTTTG |
| Human CASC1 | Forward: AGTGAAAATGAGTTCTGCTGAGG<br>Reverse: AGGTAACAGGGCCAAAGGTG |

| | |
|---|---|
| Human CD133 | Forward: ACCGACTGAGACCCAACATC<br><br>Reverse: GACCGCAGGCTAGTTTTCAC |
| Human EPCAM | Forward: CTGGCCGTAAACTGCTTTGT<br><br>Reverse: TAAAGAGCCCGCTCTCATCG |
| Human EZH2 | Forward: GATGGGAAAGTACACGGGGA<br><br>Reverse: TGCTGTGCCCTTATCTGGAA |
| Human E2F2 | Forward: ACTCGGTATGACACTTCGCTG<br><br>Reverse: ACATTCCCCTGCCTACCCACT |
| Human HEY1 | Forward: GAGTGCGGACGAGAATGGAA<br><br>Reverse: TCGTCGGCGCTTCTCAATTA |
| Human HPRT1 | Forward: CTTTGCTGACCTGCTGGATT<br><br>Reverse: CTGCATTGTTTTGCCAGTGT |
| Human NOTCH1 | Forward: GACAGCCTCAACGGGTACAA<br><br>Reverse: CACACGTAGCCACTGGTCAT |
| Human NOTCH2 | Forward: ATTGATGACTGCCCTAACCA<br><br>Reverse: CCAGCCGTTGACACATACAC |
| Human OCT4 | Forward: AGTGAGAGGCAACCTGGAGA<br><br>Reverse: GTGAAGTGAGGGCTCCCATA |
| Human SP1 | Forward: CCACCATGAGCGACCAAGAT<br><br>Reverse: GTGAGGTCAAGCTCACCTGT |

**Table S4. Primer Sequences for ChIP assay**

| Primer | Sequence (5'→3') |
|---|---|
| Human E2F2-H3K14ac/H3K9ac site | Forward: CCAAAGTCTGAAAATGAAGGGGTC<br>Reverse: TTCCAGGTGTGGGAAAGGAG |
| Human EZH2-H3K14ac/H3K9ac site | Forward: CCTCATCCTCATTCAACCCGT<br>Reverse: TGGAAAACTTGGTGAACGCC |
| Human E2F2-H3K23ac site | Forward: GGTACAGCGGTGTGTAGTAGG<br>Reverse: CGCCTTACTCGCTATGCTGC |
| Human EZH2-H3K23ac site | Forward: TTTGATTATGTCTGCTGCTGCC<br>Reverse: AACTACGAACAGTGGAAGGGT |
| Human E2F2 MOZ-binding site | Forward: GACCCAGCAGTGTATTCCCC<br>Reverse: TAGTTTGCCTCCGTCCCTCA |
| Human EZH2 MOZ-binding site | Forward: CCTCATCCTCATTCAACCCGT<br>Reverse: TGGAAAACTTGGTGAACGCC |

**Table S5. Cloning and Sequencing Primer Sequences**

| Primer | Sequence (5'→3') |
|---|---|
| BRPF1 promoter (cloning) | Forward: CGCGGCGGTACCAATAAGGAACTTAAAGAACTGAAC<br>Reverse: TATATAGCTAGCCTGGAGTTGCCCAGAGAGGTTC |
| SP1 binding site 1 mutant (cloning) | Forward: AGAAGAACAAACTCCTCACGC<br>Reverse: GCGTGAGGAGTTTGTTCTTCT |

| | |
|---|---|
| SP1 binding site 2 mutant (cloning) | Forward: TTTCGAGCCTCAGCGAACAAAC<br>Reverse: GTTTGTTCGCTGAGGCTCGAAA |
| BRPF1 (sequencing) | Forward: TGGTGGAGGAGATGGCATTT<br>Reverse: TCCTCTGACACCACATCCAG |
