# Supplementary material for: Bromodomain-containing protein BRPF1 is a therapeutic target for liver cancer
Source: Commun Biol. 2021 Jul 20;4:888. doi: 10.1038/s42003-021-02405-6 (PMC8292510; doi:10.1038/s42003-021-02405-6)
Supplement: Supplementary file 2 — Description of Additional Supplementary Files [file 42003_2021_2405_MOESM2_ESM.pdf]

Description of Additional Supplementary Files

File Name: Supplementary Data 1
Description: The source data including uncropped blots underlying Fig. 1-9 and Supplementary Fig. 1- 10 are provided as a Supplementary Data file in excel format.
