# Supplementary material for: Bromodomain-containing protein BRPF1 is a therapeutic target for liver cancer
Source: Commun Biol. 2021 Jul 20;4:888. doi: 10.1038/s42003-021-02405-6 (PMC8292510; doi:10.1038/s42003-021-02405-6)
Supplement: Supplementary file 4 — Reporting Summary [file 42003_2021_2405_MOESM4_ESM.pdf]

nature research

Corresponding author(s): Dr. Wong Chun Ming

Last updated by author(s): May 28, 2021

Reporting Summary

Nature Research wishes to improve the reproducibility of the work that we publish. This form provides structure for consistency and transparency in reporting. For further information on Nature Research policies, see our Editorial Policies and the Editorial Policy Checklist.

Statistics

For all statistical analyses, confirm that the following items are present in the figure legend, table legend, main text, or Methods section.

n/a	Confirmed	
☐	☒	The exact sample size (n) for each experimental group/condition, given as a discrete number and unit of measurement
☐	☒	A statement on whether measurements were taken from distinct samples or whether the same sample was measured repeatedly
☐	☒	The statistical test(s) used AND whether they are one- or two-sided<br>*Only common tests should be described solely by name; describe more complex techniques in the Methods section.*
☐	☒	A description of all covariates tested
☐	☒	A description of any assumptions or corrections, such as tests of normality and adjustment for multiple comparisons
☐	☒	A full description of the statistical parameters including central tendency (e.g. means) or other basic estimates (e.g. regression coefficient) AND variation (e.g. standard deviation) or associated estimates of uncertainty (e.g. confidence intervals)
☐	☒	For null hypothesis testing, the test statistic (e.g. F, t, r) with confidence intervals, effect sizes, degrees of freedom and P value noted<br>*Give P values as exact values whenever suitable.*
☒	☐	For Bayesian analysis, information on the choice of priors and Markov chain Monte Carlo settings
☒	☐	For hierarchical and complex designs, identification of the appropriate level for tests and full reporting of outcomes
☒	☐	Estimates of effect sizes (e.g. Cohen's d, Pearson's r), indicating how they were calculated

Our web collection on statistics for biologists contains articles on many of the points above.

Software and code

Policy information about availability of computer code

Data collection	N/A
Data analysis	N/A

For manuscripts utilizing custom algorithms or software that are central to the research but not yet described in published literature, software must be made available to editors and reviewers. We strongly encourage code deposition in a community repository (e.g. GitHub). See the Nature Research guidelines for submitting code & software for further information.

Data

Policy information about availability of data

All manuscripts must include a data availability statement. This statement should provide the following information, where applicable:

- Accession codes, unique identifiers, or web links for publicly available datasets
- A list of figures that have associated raw data
- A description of any restrictions on data availability

RNA-seq data from this study are available in NCBI BioProject (Accession ID: PRJNA701710, PRJNA701712, PRJNA70173 and PRJNA701714). The source data including uncropped blots underlying Fig. 1-9 and Supplementary Fig. 1-10 are provided as a Supplementary Data file. Publicly released microarray data are available via GEO (accession GSE23450 and GSE23451). RNA-seq data from in-house clinical samples can be accessed through NCBI Bioproject (Accession ID: 294031). A reporting summary for this article is available as a supplementary information file.

Field-specific reporting

Please select the one below that is the best fit for your research. If you are not sure, read the appropriate sections before making your selection.

- [x] Life sciences
- [ ] Behavioural & social sciences
- [ ] Ecological, evolutionary & environmental sciences

For a reference copy of the document with all sections, see nature.com/documents/nr-reporting-summary-flat.pdf

Life sciences study design

All studies must disclose on these points even when the disclosure is negative.

Sample size	Sample sizes were determined by ensuring enough samples for detecting statistical differences.
Data exclusions	none
Replication	Three in vivo experiments were performed on three nude mice cohorts (respective number of mice was indicated on the figure legend)<br>In vitro experiments were repeated at least three times and the attempts of replication were successful.
Randomization	For orthotopic xenograft model and subcutaneous injection model, nude mice were separated into groups based on the body weight. For the in vivo drug treatment, nude mice were separated into groups based on tumor sizes and body weight.
Blinding	Investigators were not blinded during data collection but the the difference between experimental groups is readily observable. The data was digitally analyzed at least three times in order to obtain the average.

Reporting for specific materials, systems and methods

We require information from authors about some types of materials, experimental systems and methods used in many studies. Here, indicate whether each material, system or method listed is relevant to your study. If you are not sure if a list item applies to your research, read the appropriate section before selecting a response.

Materials & experimental systems

n/a	Involved in the study	
[ ]	[x]	Antibodies
[ ]	[x]	Eukaryotic cell lines
[x]	[ ]	Palaeontology and archaeology
[ ]	[x]	Animals and other organisms
[x]	[ ]	Human research participants
[x]	[ ]	Clinical data
[x]	[ ]	Dual use research of concern

Methods

n/a	Involved in the study	
[x]	[ ]	ChIP-seq
[ ]	[x]	Flow cytometry
[x]	[ ]	MRI-based neuroimaging

Antibodies

Antibodies used

Ab Dil Vendor Catalog#
EZH2 1:1000 CST #5246
E2F2 1:1000 Santa Cruz sc-9967
a-tubulin 1:1000 CST #2148
MOZ 5ug Santa Cruz sc-293283
H3K9ac 1:1000(WB)/5ug(ChIP) CST #9649S
H3K14ac 1:1000(WB)/5ug(ChIP) CST #7627S
H3K23ac 1:1000(WB)/5ug(ChIP) Millipore 07-355
Histone H3 1:1000(WB)/5ug(ChIP) Millipore 05-928
Normal rabbit IgG 5ug Millipore 12-370
Normal mouse IgG 5ug Millipore 12-371

Validation

Validation data is available at the manufactocterer's websites with published reference and application specific data provided. No new antibodies were used in this study.

Eukaryotic cell lines

Policy information about cell lines

Cell line source(s)	Hep3B, PLC/PRF/5, HEK293FT and HEK293T were purchased from ATCC. MHCC97L was given by Dr. Z. Y. Yang from Fudan University, Shanghai, while Huh-7 was obtained from Dr. H Nakabayashi from Hokkaido University Scholl of Medicine, Japan.
Authentication	Cell lines purchased or received were authenticated using STR profiling.
Mycoplasma contamination	All cell lines were tested for mycobacteria and determined to be negative.
Commonly misidentified lines (See ICLAC register)	No commonly misidentified cell lines were used in this study.

Animals and other organisms

Policy information about studies involving animals; ARRIVE guidelines recommended for reporting animal research

Laboratory animals	Male BALB/c nude mice
Wild animals	This study did not involve wild animals.
Field-collected samples	No field collected samples were used in this study.
Ethics oversight	Animal experiments were performed with the approval of Committee on the Use of Live Animals in Teaching and Research (CULATR) of the University of Hong Kong. All experimental procedures followed strictly to the animals (Control of Experiments) ordinance of Hong Kong.

Note that full information on the approval of the study protocol must also be provided in the manuscript.

Flow Cytometry

Plots

Confirm that:

- [x] The axis labels state the marker and fluorochrome used (e.g. CD4-FITC).
- [x] The axis scales are clearly visible. Include numbers along axes only for bottom left plot of group (a 'group' is an analysis of identical markers).
- [x] All plots are contour plots with outliers or pseudocolor plots.
- [x] A numerical value for number of cells or percentage (with statistics) is provided.

Methodology

Sample preparation	For cell cycle analysis, MHCC97L cells were fixed with 70% ethanol, then treated with RnaseA and stained with propidium iodide. For apoptosis assay, MHCC97L cells were first collected, then stained with Annexin V FITC and propidium iodide.
Instrument	All analyses were performed in a BD Becton Dickinson FACSCalibur.
Software	FlowJo software (TreeStar,Asland, OR, USA) was used.
Cell population abundance	Cell populations were analyzed by gating live HCC single cell fractions. 20,000 cell counts were recorded for every sample. A representative example of flow cytometry data (dot plots) is provided in the supplementary figure.
Gating strategy	An example of the gating strategy is displayed (Supplementary Fig.5)

- [x] Tick this box to confirm that a figure exemplifying the gating strategy is provided in the Supplementary Information.
